# The Cell States of Sea Urchin During Metamorphosis Revealed by Single-Cell RNA Sequencing

**DOI:** 10.3390/ijms26031059

**Published:** 2025-01-26

**Authors:** Hui Ge, Yongyu Huang, Lili Zhang, Shiyu Huang, Guodong Wang

**Affiliations:** 1 State Key Laboratory of Mariculture Breeding, Fisheries College of Jimei University, Xiamen 361021, China; gehuizlj@163.com (H.G.); yongyuhuang10390@163.com (Y.H.); llzhang@jmu.edu.cn (L.Z.); hsy@jmu.edu.cn (S.H.); 2 Key Laboratory of Healthy Mariculture for the East China Sea, Ministry of Agriculture and Rural Affairs, Xiamen 361021, China; 3 Fisheries Research Institute of Fujian, 7 Shanhai Road, Huli, Xiamen 361000, China

**Keywords:** sea urchin, scRNA, metamorphosis, development, cell states

## Abstract

Metamorphosis is a key process in the life history of sea urchin *Heliocidaris crassispina*. However, the understanding of its molecular mechanisms is still lacking, especially the basic cell biology pre-metamorphosis and post-metamorphosis. Therefore, we employed single-cell RNA sequencing to delineate the cellular states of larvae and juveniles of *H. crassispina*. Our investigation revealed that the cell composition in sea urchins comprises six primary populations, encompassing nerve cells, skeletogenic cells, immune cells, digestive cells, germ cells, and muscle cells. Subsequently, we identified subpopulations within these cells. Our findings indicated that the larval peripheral nerves were discarded during metamorphosis. A decrease in the number of spicules was observed during this process. Additionally, we examined the differences between larval and adult pigment cells. Meanwhile, cellulase is highlighted as an essential factor for the development of competent juveniles. In summary, this study not only serves as a valuable resource for future research on sea urchins but also deepens our understanding of the intricate metamorphosis process.

## 1. Introduction

Sea urchins are widely used as model organisms to elucidate the basic mechanisms of development biology. The life cycle of *Heliocidaris crassispina* involves metamorphosis from pluteus larva to juvenile/adult, a process typical among most sea urchins. To date, a few studies have mapped the early embryonic development atlas [1,2]. To describe the pluteus structure, Smith et al. [3] have described the structure of plutei in greater detail, categorizing them into seven stages based on the number of arms. Additionally, a complementary staging scheme for the purple sea urchin (*Strongylocentrotus purpuratus*) has also been developed and found applicable to this species as well [4]. This complementary staging scheme focuses on the juvenile skeletogenic structures that form from the internal rudiment, including the interambulacral plates, spines, tube feet, and a subset of ocular plates. It is worth noting that many competent larvae, usually characterized by possessing eight arms, are capable of undergoing metamorphosis into juveniles. Here, “competent” denotes the developmental stage whereby the larva can initiate settlement and complete the morphogenic transformations associated with metamorphosis. While previous studies have defined metamorphosis as the rapid collapse of larval tissues and the growth of adult structures [5,6,7,8], this solely represents a fraction of the metamorphic process, which in sea urchins begins when the juvenile rudiment starts to develop. During the pre-metamorphosis stage, the larvae halt their ciliary beating and begin to settle onto the substrate. This is followed by the extension of primary podia from the vestibular pore, allowing them to sense their surrounding environment. Notably, settlement occurs rapidly, and the molecular mechanisms driving this process are largely influenced by exogenous signals mediated through membrane receptor systems, such as histamine and serotonin. In contrast, the subsequent developmental and morphogenic transformations rely primarily on signaling systems and hormones, including retinoic acid and thyroid hormones. In instances of unsuitable substratum conditions, the podia retract back into the vestibular pore, promoting the larvae to swim to return to the surface until the appropriate signal for settling is detected again [7,9,10]. Metamorphic morphological changes unfold rapidly, typically within a few hours. In the initial post-metamorphosis period, newborn juveniles lack the oral structure essential for feeding, and they are capable of movement, but slow [10].

The cytological processes encompassing cell differentiation, transformation, proliferation, and apoptosis form the foundation for morphological and structural alterations, highlighting the critical role of cellular changes in elucidating the molecular mechanisms of metamorphosis. Single-cell RNA sequencing (scRNA-seq) facilitates the precise evaluation of each cell state [11]. For instance, Foster et al. leveraged this technology to characterize embryonic cell states and gene expression in the sea urchin *S. purpuratus*, identifying 22 distinct cell clusters [12]. Similarly, Massri et al. examined the transcriptional changes in the cell states of sea urchin *Lytechinus variegatus*, from embryos to larvae [13]. Additionally, Perillo et al. explored the dynamic regulation of pigment cells (PCs) during the embryonic phase of *S. purpuratus*, offering valuable insights into pigmentation biosynthesis [14]. In another study, Paganos et al. delineated 21 unique cell clusters, providing detailed perspectives on the nervous system of *S. purpuratus* pluteus [15]. Despite these contributions, there is a paucity of studies focused on the specific biological processes occurring during metamorphosis, leaving the mechanisms underlying larval cell transformation during this transitional phase inadequately understood.

While the precise mechanism of metamorphosis remains elusive, it is firmly established that larval morphology starkly contrasts with that of the adult form. The primary objective of this study is to examine and compare the cell states before and after metamorphosis. The outcomes detailed herein have the potential to bridge existing knowledge gaps regarding the metamorphosis process, offering insights into the intricate molecular mechanisms and regulatory strategies at this transition.

## 2. Results

### 2.1. The Cell Atlas of H. crassispina Larvae Before and After Metamorphosis

**Figure 1:**
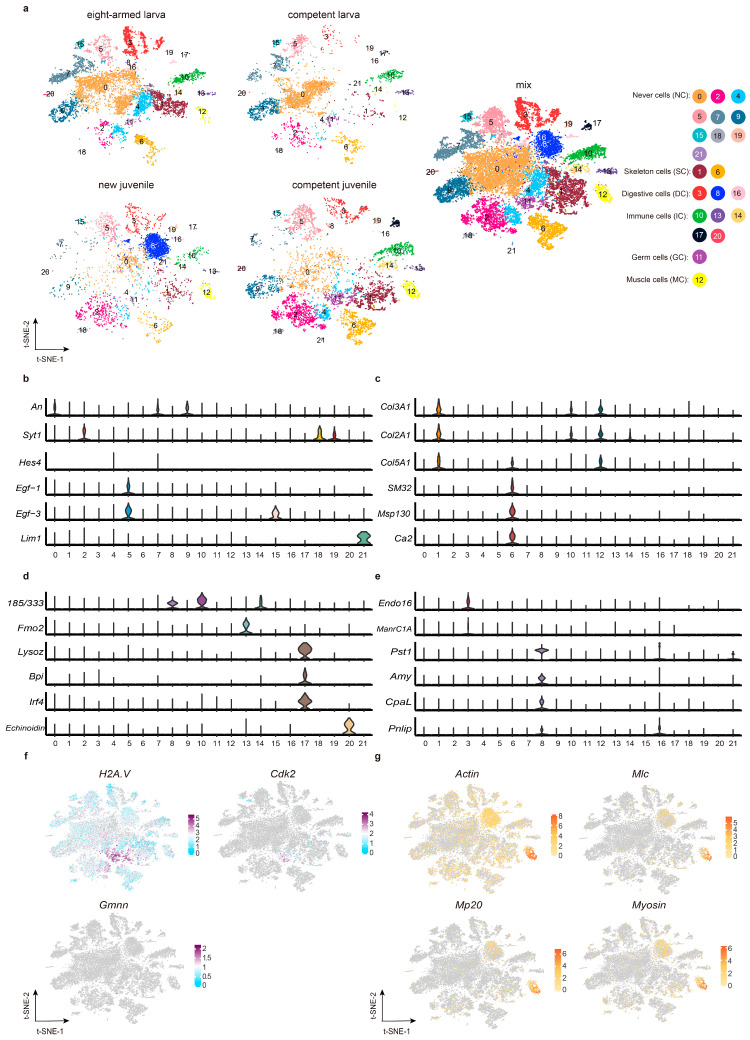
The cell atlas of the sea urchin *H. crassispina.* (**a**) t-SNE analysis was performed on cells obtained from eight-armed larvae, competent larvae, new juveniles, and competent juveniles, resulting in the identification of twenty-two cell clusters. These clusters were tagged with different colors and assigned to six groups: NCs (nerve cells), SCs (skeletogenic cells, including PMC [primary mesenchymal cell] and SMC [secondary mesenchymal cell]), ICs (immune cells, containing PCs [pigment cells, C13] and NPCs [non-pigment cells]), DCs (digestive cells), GCs (germ cells), and MCs (muscle cells). (**b**) Violin plots were utilized to depict the expression patterns of marker genes used for NC population identification. (**c**) Representative genes of the SC population are displayed using violin graphs to highlight their expression profiles. (**d**) The predominant genes of the IC group were visualized using violin plots. The violin plot represents the expression abundance of a gene in a cell cluster. The X-axis represents the cell cluster, and the Y-axis represents the expression level. (**e**) The distribution of marker genes for the DC population identification was illustrated. (**f**) t-SNE plots displayed the expression levels of marker genes used in discerning the GC population. (**g**) The identification of the MC population benefits from the expression levels of a few key genes.

Cell states were acquired from larvae before and after metamorphosis through scRNA technology. Samples included eight-armed larvae (possessing eight arms), competent larvae (can be induced to settle by KCl), new juveniles (which had just completed metamorphosis), and competent juveniles (which survived for three days and were able to feed). Scanning electron microscopy was used to visualize the developmental stages in the context of metamorphosis (Supplementary File S1). Finally, the samples yielded 9827 (eight-armed larvae), 4653 (competent larvae), 3911 (new juveniles), and 7387 (competent juveniles) cells, respectively. Figure 1a displays the 22 diverse cell clusters that were identified, each of which may contribute to our understanding of metamorphosis. Remarkably, C8 (cluster 8) exhibited high expression levels in new juveniles but was absent in competent larvae, with C21 initially emerging in competent larvae among the specified stages. The cell types within the 22 clusters were initially characterized using established marker genes, categorizing them into six groups: nerve cells (NC), skeletogenic cells (SC), digestive cells (DC), immune cells (IC; comprising pigment cells [PC] and non-pigment cells [NPC]), muscle cells (MC), and germ cells (GC) as outlined in Figure 1a.

During the initial phase of mapping the cell landscape, a crucial task was to delineate the cellular states within each cluster. The NC population was identified based on previous research highlighting an abundance of neural marker genes in *S. purpuratus* larvae [15]. In this study, ten clusters (0, 2, 4, 5, 7, 9, 15, 18, 19, and 21) were defined as NCs (Figure 1b). C0, C7, and C9 were enriched for the *An* transcript associated with the ciliary band neuron [16]. Additionally, genes abundant in neuropeptide precursors (NPs), such as *Syt1* (synaptotagmin 1) and *F-salmfa* (F-type SALMFamide neuropeptide precursor), were detected in C2 [16,17]. C4 was assigned to the NC cluster due to the similarity in *Unc-4* expression, and *Hes* (hairy enhancer of split) expression equivalent to that of C7 [15,18]. *Egf1* (fibropellin-1) demonstrated the highest expression levels in C5 and C15 [19], along with glutamate receptors. C18 showed enrichment in *Syt1* and several NP genes such as *Np9*, *Np13*, and *Cck* [20], while C19 presented genes such as *Np9*, *Np10*, *Np13*, and *F-salmfa*. C21 displayed a significant expression of *Lim1/Lmx1*, a marker gene of apical organ neurons. A genome-wide analysis of biomineralization-related proteins in *S. purpuratus* facilitates identifying skeletogenic cells. The SC population consisted of two clusters (C1 and C6) (Figure 1c). Many *Collagen* genes present high expression levels in C1 (PMC, primary mesenchymal cell), which provide the necessary substrate for spicules’ development [21,22,23]. Genes involved in mineralization, such as *SM30*, *SM32*, *SM37*, *Msp130*, *PM27* (primary mesenchyme-specific protein PM27), *P16* (biomineralization protein P16), *P19L*, and *Ca2* (carbonic anhydrase 2), were greatly expressed in C6 (SMC, secondary mesenchymal cell) [24]. The DC population consisted of three clusters (C3, C8, and C16) (Figure 1e), which either contains the midgut cell marker gene *Endo16* and *ManrC1A*, or co-presents many digestive enzyme genes, such as *PstI* (aqualysin-1-like), *Amy* (alpha-amylase), *CpaL* (carboxypeptidase B-like), and *Pnlip* (pancreatic triacylglycerol lipase) [12,14,25,26,27]. The immune cell (IC) population, distinguished by known immune genes, was subdivided into five clusters (C10, C13, C14, C17, and C20) (Figure 1d) [28], with C13 found to be a PC population, supported by the high expression level of marker genes including *Fmo2*, *Fmo5* (dimethylaniline monooxygenase [N-oxide-forming] 5), *Pks1* (probable polyketide synthase 1), *Pks19*, and *Sult1E1* (sulfotransferase) [14,29,30,31]. C11 represented the GC population, displaying markers including *H2A.V*, *Cdk1* (cyclin-dependent kinase 1), and *Gmnn* (geminin) (Figure 1f) [12,29,32]. C12 contained many genes related to muscle function, including *Actin*, *Myosin*, *Mlc* (myosin light chain), and *Mp20* (muscle-specific protein 20), serving as a distinct marker for distinguishing the MC population (Figure 1g). In summary, these findings underscore the cellular diversity in sea urchins (Further details are outlined in Supplementary File S2, while gene annotations are available in Supplementary Data S1).

To ensure the uniformity of transcript levels throughout the four developmental stages, a set of 23 genes spanning six distinct cell populations (Supplementary File S3) were chosen for qRT-PCR analysis on dissociated samples. Additionally, an investigation into the gene expression dynamics during the earlier stages, involving both four-armed and six-armed larvae, was also conducted. The detailed expression profiles of these genes are provided in the subsequent sections.

### 2.2. The Diversities of Cell Abundances Revealed Key Biological Process During the Metamorphosis

**Figure 2:**
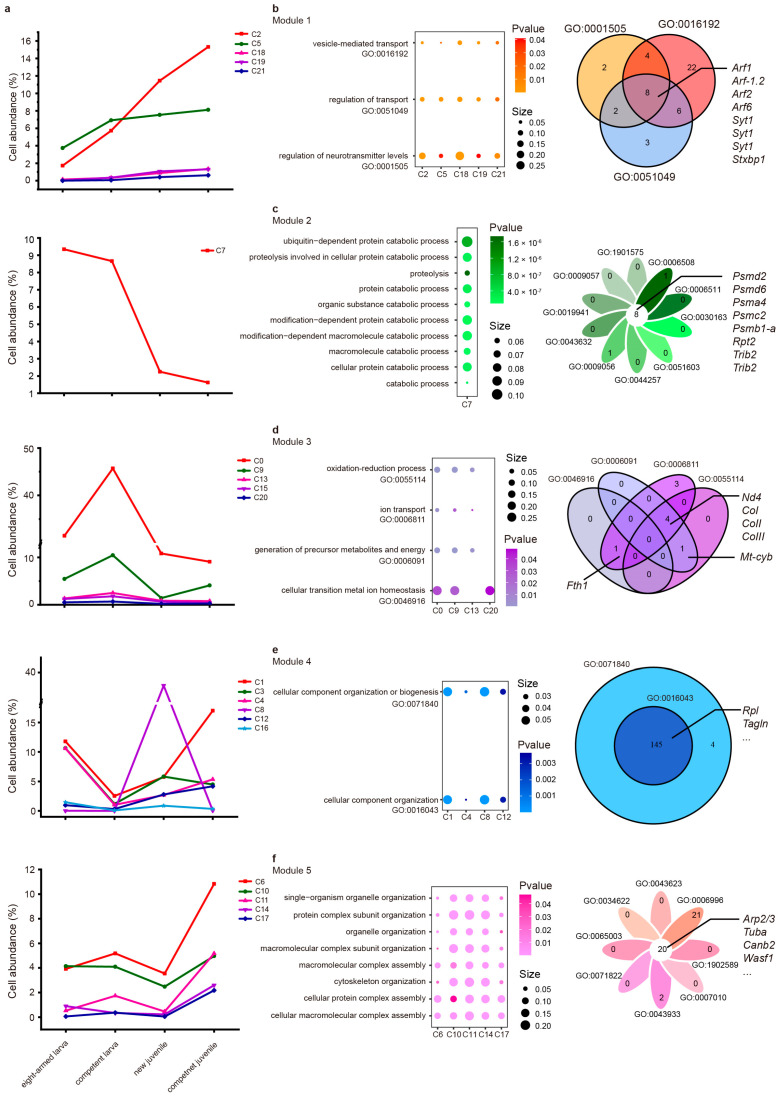
The cell cluster profiles during metamorphosis. (**a**) Line charts show the changes in cell frequency for the five modules. Each sample’s total cell abundance sums up to 100%, with the Y-axis denoting the proportion of a specific cluster within the sample. (**b**–**f**) Except for Module 2 (**c**)**,** common biological processes enriched by at least three clusters in Module 1 (**b**), 3 (**d**), 4 (**e**), and 5 (**f**) are displayed on the left. Venn diagrams visually represent the shared upregulated genes within each GO in terms of different clusters on the right. It should be noted that genes with the same name represent different transcripts.

Heterogeneity in cell frequency across different clusters during metamorphosis was observed. To explore the link between cell abundance and biological processes, all clusters were categorized into five modules (Figure 2a). Module 1, comprising C2, C5, C18, C19, and C21, demonstrated an escalating cell frequency trend. Module 2, represented by only C7, exhibited a decline in cell proportion. Module 3 showcased the highest cell abundance in the competent larva, encompassing C0, C9, C13, C15, and C20. Module 4, composed of C1, C3, C4, C8, C12, and C16, displayed the lowest cell abundance in the competent larva. Lastly, Module 5 included C6, C10, C11, C14, and C17, with the lowest cell frequencies in the new juvenile and the highest in the competent juvenile. GO analysis was employed to investigate the biological processes among the upregulated genes in each module. For more comprehensive information, please refer to Supplementary Data S2.

Figure 2b demonstrates the shared biological processes within Module 1, which primarily contained “regulation of neurotransmitter levels”, “vesicle-mediated transport”, and “regulation of transport”. The key genes enriched in these processes included *Arf1* (ADP-ribosylation factor 1), *Arf2*, *Arf6, Stxbp1* (syntaxin-binding protein 1), and *Syt1*. These findings imply a high level of activity in neurotransmitter transport during metamorphosis.

Module 2 (Figure 2c) highlighted that C7 was notably enriched in catabolic processes, including “organic substance catabolic”, “macromolecule catabolic”, and “protein catabolic”. Genes associated with these processes comprised *Psm* (proteasome), *Trib2* (tribbles homolog 2), and *Rpt2* (proteasome 26S subunit subunit 4 ATPase-like). These results suggest a potential involvement of catabolism in the metamorphic process.

Module 3 failed to display any shared biological processes among its clusters (Figure 2d). Consequently, our focus shifted toward processes that were collectively represented in at least three clusters. For instance, the processes of “cellular transition metal ion homeostasis” were common among C0, C9, and C13, while C0, C13, and C20 shared functions such as “oxidation–reduction processes”, “generation of precursor metabolites and energy”, and “ion transport”. Genes involved in these processes, including *Nd4*, *CoI*, *CoII*, *CoIII*, *Fth1* (soma ferritin), and *Mt-cyb* (cytochrome b), were visualized. These results hint that the upregulation of ion transport plays a vital role in metamorphosis.

Module 4, similar to Module 3, lacked in exhibiting shared biological processes among its clusters (Figure 2e). Hence, we explored processes enriched in the four clusters (C1, C4, C8, and C12), including “cellular component organization or biogenesis” and “cellular component organization”. Module 5 focused on intersectant processes such as “cellular protein complex assembly”, “cellular macromolecular complex assembly”, “macromolecular complex assembly”, “protein complex subunit organization”, “macromolecular complex subunit organization”, “cytoskeleton organization”, “single-organism organelle organization”, and “organelle organization” (Figure 2f). The genes enriched in these processes included *Arp2/3*, *Tuba*, *Canb2*, and *Wasf1*. These results suggest that the assembly of macromolecular complexes plays a fundamental role in the cytoskeleton construction of juvenile organisms.

In summary, modules were constructed based on variations in cell frequency, irrespective of the cell states in each cluster. Identifying common processes among these clusters may enrich our understanding of the metamorphosis process.

### 2.3. WGCNA Analysis of the NC Population

Gene co-expression networks facilitate the recognition of hub genes within distinct clusters. Utilizing WGCNA (weighted gene co-expression network analysis), we established 21 modules labeled with unique colors (Supplementary File S4, Figure 1). To identify hub genes in the NC cluster, we selected a representative subcluster for detailed analysis.

Interestingly, C21 was initially detected in the competent larva among the four stages, suggesting its potential as a pivotal cluster during metamorphosis. The qRT-PCR results show that although the marker gene *Lim* is expressed at early larval stages, its expression significantly increases in the competent larva (Supplementary File S3). The GO enrichment analysis of the blue module revealed a focus on metabolic and biosynthetic processes (Supplementary File S4, Figure 2a). Key metabolic processes mainly included the “nitrogen compound metabolic process”, “primary metabolic process”, “heterocycle metabolic process”, and “cellular metabolic process”. In terms of biosynthetic processes, the ‘cellular nitrogen compound biosynthesis process’ and ‘organic substance biosynthesis process’ were representative items. Nitrogen compounds appeared to play a significant role within this cluster. Further examination identified *Pipox* (peroxisomal sarcosine oxidase-like), *Trabd* (traB domain-containing protein), *Ndufa2* (NADH dehydrogenase [ubiquinone] 1 alpha subcomplex subunit 2-like), transcription factor *Lim1*, and *Mmadhc* (methylmalonic aciduria and homocystinuria type D homolog, mitochondrial) as potential key genes (Supplementary File S4, Figure 2b).

### 2.4. Regularity of Changes in Cell Frequency of NC Subsets

Each cluster may encompass cell states of both larva and juvenile. Thus, we endeavored to assess their cellular differences through an examination of the NC subsets.

**Figure 3:**
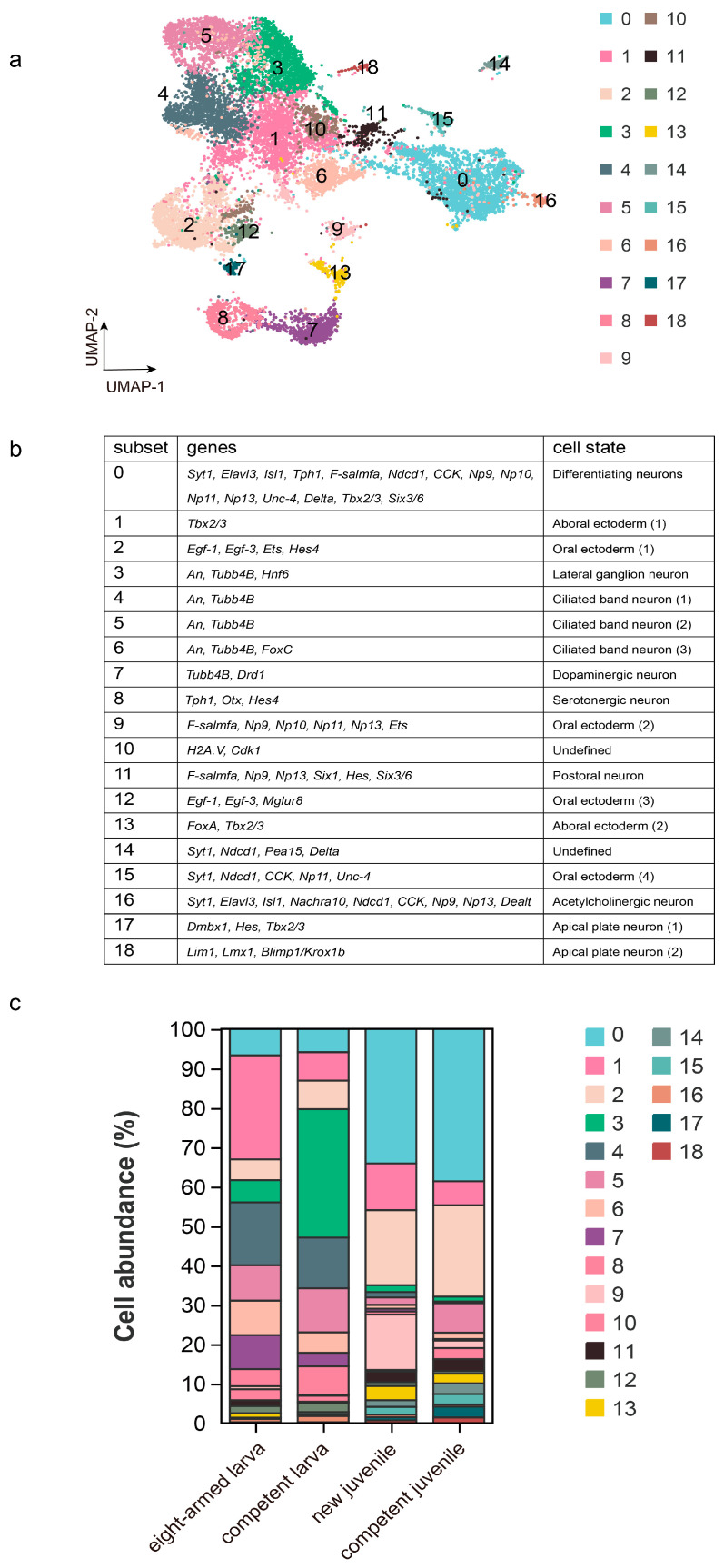
The cellular heterogeneity in the NC subsets. (**a**) The UMAP graph shows the NC subsets at a resolution of 0.5. (**b**) The marker genes used to identify subsets. (**c**) The cell abundances of 19 NC subsets.

A total of 19 subsets were observed by subdividing the NC population (Figure 3a) (See the Supplementary File S5 for details). The marker genes used are listed in Figure 3b. Our analysis concentrated on the expression patterns of two genes: *An*, linked to the ciliary band, and neuropeptide *F-salmfa*, found in the rudiment [33], which is the core structure of forming juveniles. Subsets 3, 4, 5, and 6 highly expressed *An*, with their cell frequencies declining by 2.73% to 30.84% from the competent larva to the new juvenile. Conversely, subsets 0, 9, and 11 demonstrated elevated levels of *F-salmfa*, showing respective increases in cell abundance of 28.18%, 13.74%, and 2.38% from the competent larva to the new juvenile (Figure 3c).

Additionally, the abundance of oral neurons and apical neurons also increased. The apical organ serves as the central nerve organ in larvae, contributing to substrate selection and metamorphosis regulation. The apical neuropile and oral ganglion may act as nerve centers to regulate signal perception and control the beginning of metamorphosis [34]. Our results showed an elevation in their cell abundance throughout metamorphosis, while the nerves associated with the ciliary band exhibited a decrease. We found that the expression of the adult *An* gene decreased sharply compared to the competent larva. Furthermore, the expression level of *F-salmfa* significantly increases between competent larva and the new juvenile, while *Lim* also maintains a high level in the new juvenile (Supplementary File S3). This implies a significant role of larval nerves in the metamorphic process.

### 2.5. WGCNA Analysis Revealed the Importance of Hydrogen Transport in C6 of the SC Population

C6 stood out as a prominent cluster within the SC population, showcasing the expression of numerous biomineralization-related genes. The GO analysis of the dark red module exhibited dominant terms such as “hydrogen transport” and “generation of precursor metabolites and energy” (Supplementary File S4, Figure 3a). The co-expression network mined several candidate genes, including *Pla2* (phospholipase A2 AP-PLA2-I, PLA2), *Slc4a10*, *P16*, *Tspan4* (tetraspanin-4), *SM29*, *SM30*, *Srcr* (scavenger receptor cysteine-rich domain superfamily protein), and *LOC115920494* (Supplementary File S4, Figure 3b). *Slc4a10* may be a potential marker with vital importance for the larval calcification under acidification conditions [35,36,37]. Its abundance is highest at competent larva (Supplementary File S3). The data indicate that hydrogen transport plays a critical role in mineralization.

### 2.6. Different SC Subclusters Enriched Different Matrix Proteins

**Figure 4:**
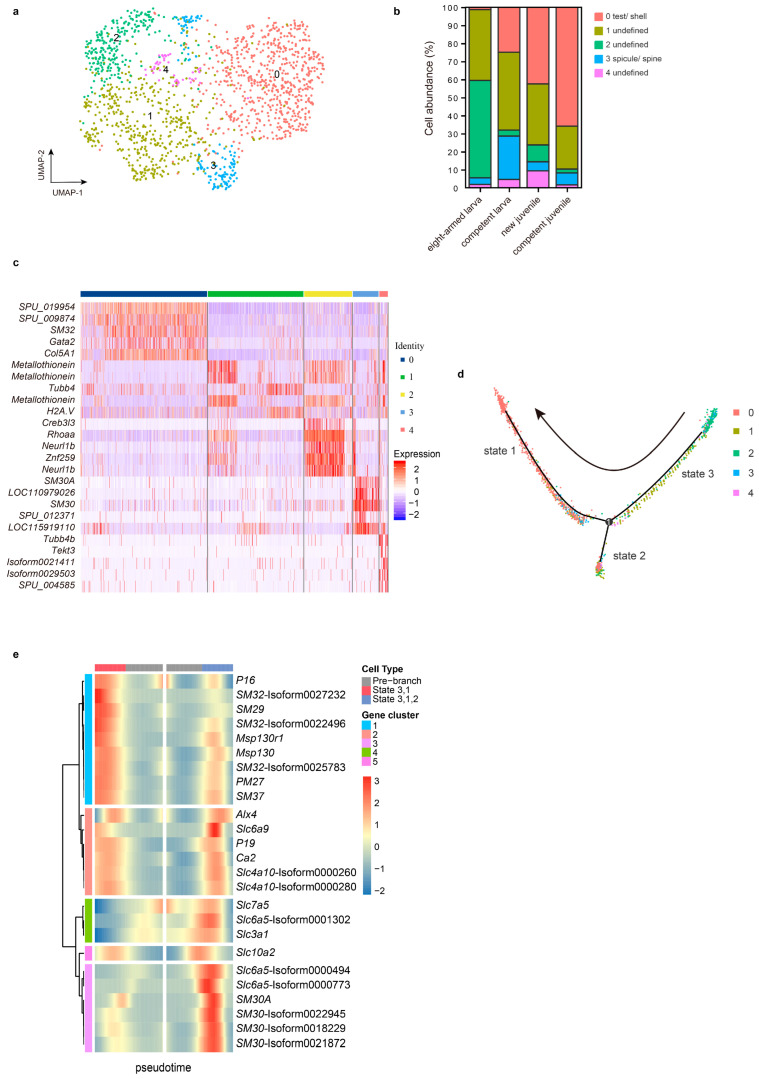
Cell classification of C6 in the SC population. (**a**) The UMAP plot illustrates the C6 subclusters at a resolution of 0.5. (**b**) The cell frequencies of the four subclusters. (**c**) The heatmap displays the top five upregulated genes (based on fold change) in each subcluster. (**d**) The distribution of the four subclusters along the pseudotime trajectory. (**e**) The distribution and clustering of symbolic genes with different differentiation fates. Note: “Isoform” means unknown; genes sharing the same name represent different transcripts; the “SPU” genes can be found in the Echinobase (https://www.echinobase.org/entry/, accessed on 20 April 2022).

SMCs mainly participate in skeletogenic formation in both larvae and adults. First, an interesting question is which cells in the SMC population develop into adult skeletogenic cells. Additionally, do the adult spines and test have regulation by the same matrix proteins? Figure 4a shows that C6 comprises five distinct subclusters, while Figure 4c illustrates different biomineralization-related genes concentrated in subclusters 0 and 3. Subcluster 0 highly expressed *SM32*, *SM29*, *PM27*, *SM37*, and *P19L*, whereas subcluster 3 significantly enriched *SM30*, *P16*, and *Ca2* (Supplementary File S6). Moreover, the cell abundances in these subclusters manifest distinct trends (Figure 4b). Notably, the cell frequency of subcluster 2 drastically decreases between eight-armed larvae and competent larvae.

To investigate the relationships among the five subclusters, we conducted pseudotime (cell trajectory) analysis to examine their evolutionary characteristics (Figure 4d). Notably, subcluster 0 displayed a distinct distribution on a separate branch compared to the other subclusters. Subcluster 2 exhibited enrichment primarily in the early stage, indicating its position at the initiation point of the pseudotime axis, although its cellular state appeared perplexing. Subcluster 3 was predominantly concentrated on a shorter branch. Here, we proposed that subcluster 0 is related to the adult skeletons, while subcluster 3 is associated with spicules or spines. This hypothesis is supported by the high expression of SM30 in subcluster 3, coupled with a 50% decrease in cell abundance during metamorphosis [38]. In the competent larva, the expression of *SM30* is significantly higher than that of early-stage larva. Meanwhile, from competent larvae to new juveniles, it shows a significant downward trend (Supplementary File S3). As the larval skeleton is shed during metamorphosis, SM30 mRNA, predominantly expressed in spicules, governs their growth [39,40,41]. Furthermore, the cell frequency of subcluster 0 continues to increase and exists at the eight-armed larva stage. These cells potentially play a role in rudiment formation, which gradually progresses in the mid and late stages of the eight-armed larva, or even earlier, particularly if larvae are well nourished and initiate rudiment formation during the six-armed stage [4,42]. As rudiment development commences, other skeletogenic structures will also form.

As previously mentioned, genes involved in mineralization appeared to be categorized into two distinct groups with varying functions. To explore further, we analyzed their expression patterns across different differentiation fates (Figure 4e). In contrast to other matrix genes, *SM30* exhibited a unique expression pattern characterized by an initial increase followed by a decrease in state 3. Conversely, *SM29*, *SM32*, *PM27,* and others displayed heightened expression toward the end of the pseudotime axis in state 1. Interestingly, the expression profiles of several *Slc* genes also suggest their involvement in the mineralization process, especially the *slc6a5* gathered with *SM30*, and the *slc4a10* identified in WGCNA. They show the highest abundance in the competent larva (Supplementary File S3).

### 2.7. The Gene Co-Expression Network of PC Was Revealed by WGCNA Analysis

To identify important processes and key genes relevant to PC, we focused on the magenta module of C13. This module exhibited enrichment primarily in “multi-organism behavior”, “nucleotide transport”, and “defense response” (Supplementary File S4, Figure 4a). The gene network implied that *sult1E1*, transcription factor *hox-d9a*, *pks1*, *fmo5*, *tmem163* (transmembrane protein 163), and *protein D3* serve as hub genes (Supplementary File S4, Figure 4b). These genes may be involved in pigment formation or defense response. Particularly, transcription factor hox-d9a may be a potential marker.

### 2.8. The Molecular Characteristics of PC Subgroups in Larva and Juvenile

**Figure 5:**
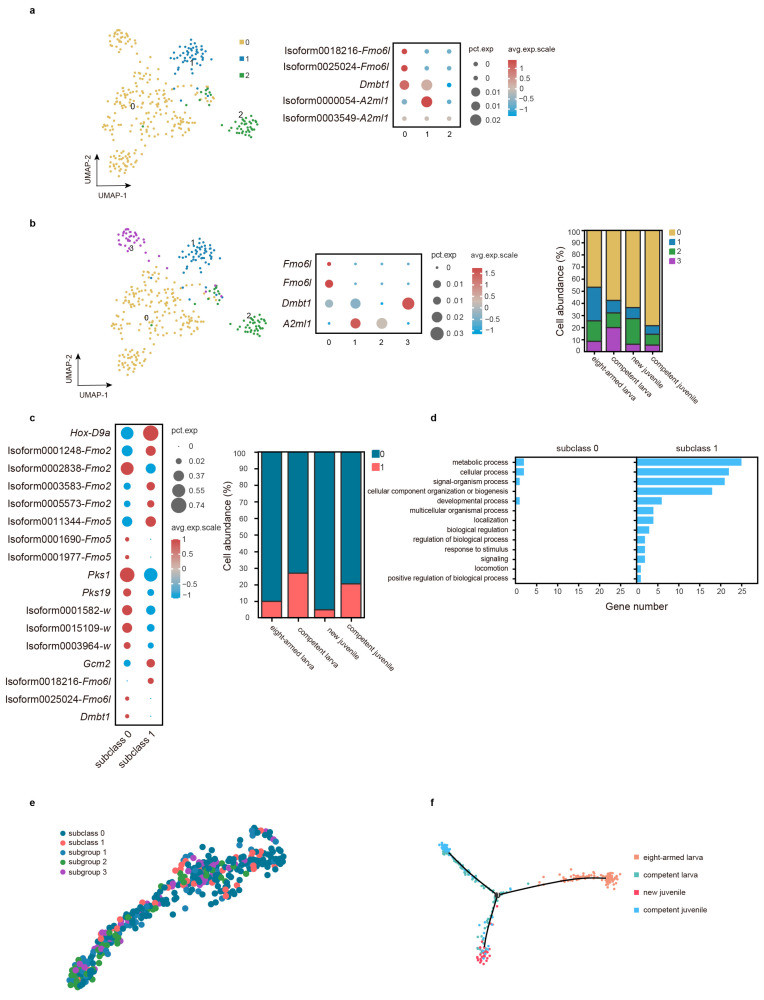
The overall landscape of C13 in PC. (**a**) The UMAP graph represents the C13 subgroups at a resolution of 0.2 (left), while the bubble graph displays the expression levels of *Dmbt*, *A2ml1*, and *Fmo6l* in each subgroup (right). (**b**) The UMAP graph portrays the C13 subgroups at a resolution of 0.5 (left); the bubble graph shows the expression levels of *Dmbt*, *A2ml1*, and *Fmo6l* in each subgroup (middle), alongside the cell abundances of the four subgroups (right). (**c**) The bubble graph demonstrates the expression profiles of marker genes in two subclasses of subgroup 1 at a resolution of 0.8 (left), accompanied by the cell frequencies of the two subclasses. (**d**) A comparison of GO terms between the two subclasses. (**e**) The distribution of these subclusters on the pseudotime trajectory using PAGA analysis. (**f**) The pseudotime trajectory using Monocle based on samples. Note: “Isoform” means unknown, and genes with the same name represent different transcripts.

The purple sea urchin *H. crassispina* is a suitable model organism to scrutinize the PC genesis and pigment synthesis. The genes *Sp-srcr142* and *Sp-tecp2* are specifically expressed in the larval PCs of *S. purpuratus* [43]. However, not all larval PCs express both transcripts simultaneously, and they are absent in adult PCs. For comparison to the PC of *S. purpuratus*, we identified the homologous genes of *Sp-srcr142* and *Sp-tecp2*, which are *Dmbt1* (Isoform0000245) and *A2ml1* (Isoform0000054, Isoform0003549), respectively. Next, we divided C13 into three subgroups with a ’resolution’ parameter of 0.2, which generated a subgroup (subgroup 2) that expressed neither of these two genes (Figure 5a). This indicates that subgroup 0 and subgroup 1 represent larval PCs, while subgroup 2 represents adult PCs. Nevertheless, as *Fmo6l* is also expressed in adult PCs (Supplementary File S3), this categorization may have limitations [14].

Subsequently, the resolution was increased to 0.5, and higher resolutions led to a greater number of subgroups (Figure 5b). Increasing the resolution facilitated the identification of various cell types. In this case, *dmbt1* was found in subgroups 0, 1, and 3, whereas *A2ml1* was detected in subgroups 1 and 2. Surprisingly, the subgroup that failed to express those two genes was missing. Moreover, *Fmo6l* remained exclusively expressed in subgroup 0 (Figure 5b). As *Fmo6l* is expressed in both larval and adult PCs (Supplementary File S3), subgroup 0 may encompass both larval and adult PCs. To explore the heterogeneity of subgroup 0, it was further divided into subclass 0 (*Dmbt1*^+^ and *Fmo6l*^+^) and subclass 1 (*Fmo6l*^+^) (Figure 5c). Various markers were analyzed in these subclasses, revealing that two *Fmo5* transcripts were exclusively expressed in subclass 0. Furthermore, the frequency of subclass 1 increased in juveniles, while that of subclass 0 decreased (Figure 5c). These findings indicate potential functional differences between the two subclasses. Subsequently, biological processes were compared and visualized, with Figure 5d displaying a greater enrichment of subclass 1 in items such as “biological regulation” and “response to stimulus”.

Pseudotime analysis was implemented to investigate the differentiation relationship between larval and adult cells. Figure 5e demonstrates that adult PCs may not have directly differentiated from larvae. Surprisingly, the pseudotime axis, constructed from the samples, positioned the new juvenile and competent juvenile in separate branches, indicating distinct cellular states (Figure 5f).

In conclusion, our study revealed that PCs might exhibit heterogeneous expression patterns of *Dmbt1* and *A2ml1*, which were similar to those of *S. purpuratus*. However, notably, qRT-PCR results show that they are expressed in both larvae and juveniles (Supplementary File S3). Furthermore, *Fmo6l* was also detected in larval and adult PCs of *H. crassispina*, aligning with its expression in *S. purpuratus* [14].

### 2.9. Many Digestive Enzymes in C16 of the DC Population Were Revealed by WGCNA Analysis

In the DC population, C8 was notable; however, it failed to align with any significant module. Consequently, C16, which also demonstrated high expression levels of digestive enzymes, was chosen to identify key genes. An examination of the purple module unveiled pertinent terms such as “protein modification by small protein conjugation or removal” and “mRNA metabolic process” (Supplementary File S4, Figure 5a). The gene network highlighted *Amy*, *Pnlip*, *Gnbp1* (beta-1,3-glucan-binding protein), *Cpal*, *Cellulase*, and *PstI* as hub genes (Supplementary File S4, Figure 5b).

### 2.10. The Complex and Variable DC Subpopulations

**Figure 6:**
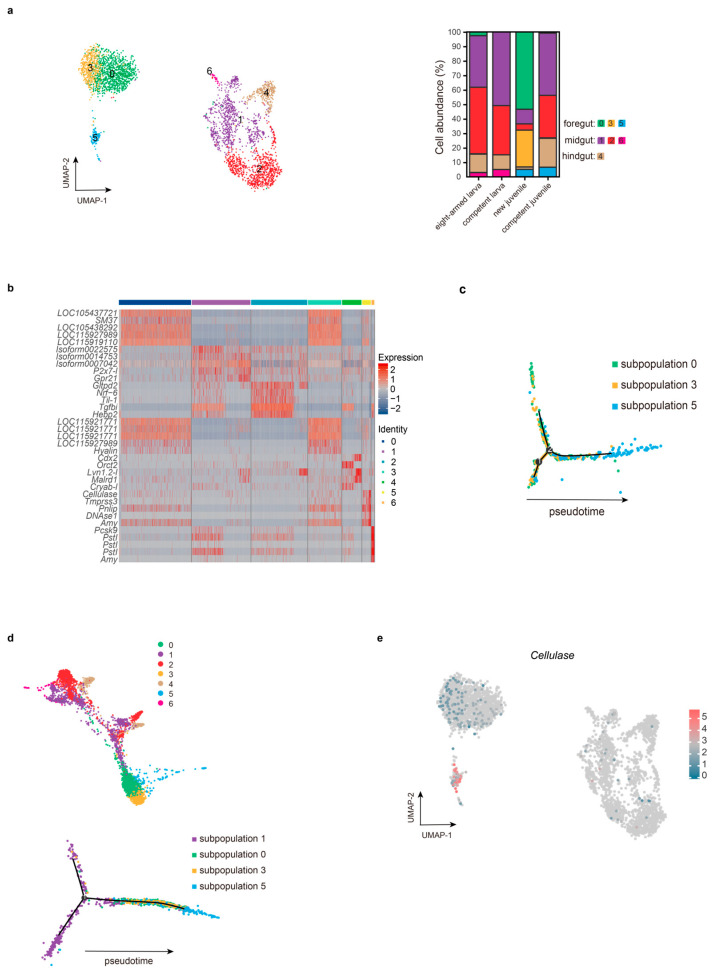
The overall landscape of the DC population. (**a**) The UMAP graph illustrates the distribution of the DC population at a resolution of 0.5 (left) and the cell abundances of the seven subpopulations (right). (**b**) The heatmap displays the top five upregulated genes of each subpopulation based on fold change. (**c**) The distribution of subpopulations 0, 3, and 5 on the pseudotime trajectory. (**d**) PAGA analysis was utilized to display the distribution of the seven subpopulations on the pseudotime trajectory (above); the pseudotime trajectory of subpopulations 0, 1, 3, and 5 (below). (**e**) The distribution of *cellulase* on the UMAP plot. Note: “Isoform” means unknown, and genes with the same name represent different transcripts.

To investigate alterations in the digestive system, we categorized the DC population into seven subgroups: three foregut cells (0, 3, 5), three midgut cells (1, 2, 6), and a hindgut cell (4) (Figure 6a). Noteworthy, it is essential to note that these subgroups may not uniformly apply to both larvae and juveniles, given that the larval foregut may not necessarily correspond to the juvenile foregut. The C8 formed the majority of subpopulations 0, 3, and 5 here, as they were non-existent in the competent larva (Figure 6a). To expand our findings, we analyzed the upregulated genes across all subgroups (Figure 6b), revealing the heightened expression of several mineralized genes, such as *SM37*, *P19*, and *SM32*, in subpopulations 0, 3, and 5. In fact, the digestive organ expresses calcareous ossicles, which are secreted by scleroblasts in the wall of the digestive system, developed in the rudiment [10,44]. These ossicles prevent sharp food from stabbing the wall. Although the main cell types of the digestive system have been described, it remains challenging to identify them [44].

Furthermore, both subpopulations 5 and 6 exhibited elevated expression levels of various digestive enzymes. While classified as exocrine cells, only subpopulation 5 persisted into the competent juvenile stage (Figure 6a). Two interesting events arose from the analysis of these subpopulations: (i) How did subpopulations 0, 3, and 5 abruptly appear in large numbers at the new juvenile stage? Could it be related to the decreased cell abundance of subpopulations 1, 2, and 4? (ii) What factors contribute to the diametrically opposite fates of subpopulations 5 and 6?

Regarding the first event, we explored the connection among subpopulations 0, 3, and 5 (Figure 6c). Due to more cells in subpopulation 5 of competent juveniles, only subpopulation 5 enriched many digestive enzymes. Thus, the differentiation degree of subpopulation 5 was higher than subpopulations 0 and 3. Next, PAGA analysis was carried out to investigate the differentiation relationship between subpopulations 0 or 3 and other subpopulations (1, 2, 4, and 6). These findings showed that only subpopulations 1 and 0 were interconnected, indicating that subpopulation 1 might differentiate into subpopulation 0 (Figure 6d). Therefore, a pseudotime ordering of subpopulations (0, 1, 3, and 5) was established, revealing a trajectory with three branches (Figure 6d). It revealed that subpopulation 1 mainly distributed into two branches, while subpopulations 0, 3, and 5 appeared on another single branch. This suggests that subpopulation 1 differentiated into subpopulations 0 and 3, which eventually gave rise to subpopulation 5.

In the second event, we compared the digestive enzyme types present in subpopulations 5 and 6. Our analysis revealed that *PstI*, *Amy*, *Cpal*, and *Pnlip* were upregulated in both subpopulations. Next, the difference in cellulase was also examined because cellulose is the main component of the plant cell wall. Interestingly, one of the *cellulase* genes was highly accumulated only in subpopulation 5 rather than subpopulation 6 (Figure 6e). The qRT-PCR results show that it expressed at all stages, and the level of competent juvenile is significantly higher than that of new juvenile (Supplementary File S3). Currently, cellulase has been identified in the digestive organs of sea urchins [45]. These organisms utilize digestive enzymes to break down algal cell walls and membranes, particularly in macroalgae comprising cellulose and alginic acid [46]. To conclude, these outcomes provide valuable insights into the adaptive adjustments made by competent juveniles.

## 3. Discussion

Previous studies have reported the cells at the early developmental stage of sea urchin *S. purpuratus* and *Lytechinus variegatus* [12,14,15]. However, the available scRNA data of juveniles or adults are still lacking. Our objective was to study the cell states of *H. crassispina* both pre- and post-metamorphosis with the intention of uncovering insights that could aid in comprehending the molecular processes underlying metamorphosis.

The metamorphosis process of marine invertebrates is closely associated with changes in the nervous system, including cell death, reshaping, the merging of nerve cells, and the differentiation of new cells [47,48,49]. For example, in mollusks, the larval velum, a specialized structure, is discarded during metamorphosis along with its associated neurons, while other neurons are retained and utilized in forming the adult nervous system [47,48,49,50]. This study revealed that the peripheral nervous system in sea urchin larvae is largely discarded during metamorphosis, notably exemplified by the removal of ciliary band neurons crucial for larval swimming and feeding activities, utilizing cilia movements. However, with the transition to a benthic diet in adults, the maintenance of cilia becomes dispensable. An examination of the cell expression frequency of *An* (Subsets 3, 4, 5, and 6) revealed a significant decline during metamorphosis. The expression of the adult *An* gene decreased sharply compared to the competent larva (Supplementary File S3). Additionally, it was suggested that some larval central nervous system components persist into the juvenile stage. The rise in cell frequency during metamorphosis predominantly involved neurons associated with the apical neuropile and oral ganglion, which are believed to mediate natural cue perception and regulate the initiation of metamorphosis [34]. The qRT-PCR also shows that the expression level of F-salmfa significantly increases between competent larvae and new juveniles, while *Lim* also maintains a high level in new juveniles (Supplementary File S3).

Earlier studies have proposed that the larval and adult nervous systems of sea urchins functioned independently, and the apical ganglion of echinoplutei failed to control the juvenile primary tube feet [33,51,52,53]. Uncertainties arise regarding the larval settlement process and the mechanism by which it decides to swing cilia again. Do the nerves of the adult rudiment guide the larval cilia, or does the larva directly innervate the adult tube feet? Here, we propound that their nerves are partially integrated during metamorphosis. However, it is crucial to note that this proposal is purely speculative and not substantiated by our data, and our results are insufficient to provide evidence. Indeed, the apical ganglion is connected to the adult rudiment via fibers [33], suggesting some level of coordination between larval and adult nervous systems. If 5-HT is involved in settlement, it may be part of the pathway integrating the larval and juvenile nerves [33]. Furthermore, the larval nerves may serve as a leading role. For example, the elimination of the ectoderm containing the post-oral ciliary band can hinder competent larvae from responding to biofilms, leading to a delay in the onset of metamorphosis. Moreover, the post-oral ciliary band may convey the biofilm signal by reducing the output of NO signals. Consistent with sensory inputs from the tube feet of the juvenile, the reduction in NO/cGMP signaling may mediate the collapse of the larval body and initiate metamorphosis [54,55]. This emphasizes the significant role of nitrogen compounds during metamorphosis. In this study, we found that the “nitrogen compound metabolic process” is important for C21 and have further identified the involvement of *Nosip* (nitric oxide synthase-interacting protein) and GMPs (GMP synthase) in this process. This may highlight the importance of larval nerves and may explain the low survival rate of juveniles in natural or artificial culturing. Once several conditions for metamorphosis are prepared, the larva will autonomously activate the metamorphosis program, accomplishing its “mission”, even if the rudiment remains immature. There is a pressing need for more precise and refined methodologies to solve this intricate puzzle.

During metamorphosis, the spicules shift to the oral surface and are eventually discarded [38]. In this study, subcluster 3 significantly enriched *SM30*. Moreover, subcluster 3 showed the largest decrease in cell frequency during metamorphosis (−50.71%), and its expression level also significantly decreased (Supplementary File S3). This also supports the notion that larval spicules are discarded. Although many matrix proteins affect skeletogenic growth, their localization and functions are discrepant [24,41]. For instance, SM30 and SM50 interact differently with mineral components during skeleton formation [56]. Additionally, the expression patterns of SM37 and PM27 were similar to SM50, suggesting that SM30 performs a distinct role from SM50, SM37, and PM27 [39]. Furthermore, SM30-D and –F were rarely expressed in embryos, and SM30 mRNA was absent in the adult test but enriched in spines [24,39,41,57]. SM30-A is exclusively present in the spicule. In our research, SM30-A was highly expressed in subcluster 3. Gosselin et al. noted that the test diameter scarcely increases from a new juvenile to a competent juvenile, whereas the spines proliferate in number [10], implying that the test and spines are regulated by diverse matrix proteins. Therefore, we infer that SM30 is responsible for regulating spicules/ spines (subcluster 3), while the SM50 protein family primarily impacts the test.

Additionally, two SLC family genes, *slc4a10* and *slc6a5*, may play important roles in mineralization. During the early embryonic skeletogenesis stage, the *slc4a10* gene exhibited the highest expression level compared to other *slc4* transporters [36,38]. Larvae are equipped with a pHi regulatory mechanism that enhances biomineralization. The knockdown experiment of the *slc4a10* gene highlighted its essential role in regulating pHi in PMCs [36]. As the expression level of *slc4a10* gradually decreases, cells expressing slc4a10 migrate toward the tip of the spicules. In addition, *slc4a10* may also participate in the HCO3^-^ concentration mechanism, contributing to intracellular calcium accumulation, crucial for larval calcification. The accumulation of intracellular bicarbonate and carbonate ions is known to facilitate larval calcification [37]. In summary, *slc4a10* plays a key role in regulating the formation of amorphous calcium carbonate in sea urchin larval cells, particularly crucial for larval calcification under acidification conditions. Slc6a5, as a sodium- and chloride-dependent glycine transporter, remains unclear in its function in biomineralization. Nonetheless, given its close clustering with *SM30*, it seems likely to fulfill a comparable role to that of *SM30*.

Here, we posit that PCs expressing *Fmo6l* can be categorized into two types (*Fmo6l*^+^, and *Fmo6l*^+^ + *Dmbt1*^+^), yet its mode of action on PCs remains unclear. Pigmentation might just be one of the diverse functions of PCs [14]. The pesudotime analysis implies that new juveniles and competent juveniles encompass different types of PCs. Additionally, we observed an increase in the abundance of subclass 1 in competent juvenile. Sea urchins are required to express a greater number or types of immune cells before developing a mouth to enhance their immune system to cope with more complex environments. Adult PCs may have two functions: immunity and pigmentation. Firstly, different Fmo enzymes are expressed in spines, tube feet, and coelomic cells, contributing to pigment synthesis [14]. Secondly, larval PCs have functions in the innate immune defense system [58,59]. Similarly, in the absence of pigments in adults, resistance to environmental challenges may be weakened [60]. Finally, pigments released by PCs directly damage microorganisms [14,61]. Overall, several studies support the role of pigments in anti-microbial activity [61,62]. We discovered that the larval PCs failed to differentiate into adult PCs. So how did adult PCs originate? A study pointed out that a group of stem cells in early embryos may supply adult PCs [14]. These stem cells may be preserved in the larva, serving as a repository for adult PCs [60]. While our study supports this hypothesis, the exact timing of the emergence of adult PCs remains unknown, representing a limitation of our research. Nonetheless, our findings indicate that adult PCs are present, at least in the eight-armed larval stage.

New juveniles need to assemble diverse macromolecular complexes to construct both a cytoskeleton and matrix proteins. As mentioned in the Introduction Section, the new juvenile cannot ingest benthic food [10,63], resulting in significant pressure on their nutrition and energy levels. This raises the question of how larvae successfully transition into competent juveniles. The larval nutrient store emerges as a critical factor, as new juveniles almost solely rely on the larval reserve [64]. This is traceable at least from the eight-armed larva; the lipid vesicles continued to accumulate until metamorphosis [3]. Lipid storage can be found in the stomach of juveniles, and the reduction in juvenile volume without adding external nutrients also reflects the consumption of lipid vesicles [63,65]. In this research, C8 was expressed in the new juvenile in large quantities, indicating its utilization of stored nutrients. Furthermore, the presence of cellulase suggests preparation for future predation on large algae. Subpopulation 5 is the main digestive cell for the competent juveniles. Here, we propose a hypothesis that upon structural maturation, larvae sense the level of nutrient accumulation via specific pathways. Upon reaching a threshold, larvae may successfully undergo metamorphosis triggered by specific signals. For instance, *FoxO* may serve as an effective biomarker for indicating nutritional stress [63]. Thus, metamorphosis is an easy process to achieve. However, the most critical aspect after metamorphosis is whether the new juvenile survives until it forms a mouth. In nature, larvae have a mortality rate of up to 90% in the first week after metamorphosis (from new juvenile to competent juvenile) [63,66]. The insufficient nutrient store is a possible factor hindering systematic development completion. Therefore, it is particularly critical to strengthen the nutrient accumulation for the eight-armed larvae. Mos et al. concluded that the survival rate of juveniles is weakly related to those signals that highly induce metamorphosis [67]. Thus, studying the role of larval nutrition in new juveniles may be a way to improve their survival rate, which helps us to eliminate the bottleneck for efficient sea urchin culturing [67].

## 4. Materials and Methods

### 4.1. Adult Sea Urchin Culturing

Adult male and female sea urchins *H. crassispina* were obtained from Dongshan Bay in Fujian Province, China. They were temporarily reared in net cages with a water temperature of 28 °C. Sea tangle and oysters were fed adequately (5% of the body weight) once a day, and any remaining feed was removed the next day. Gamete release was induced by injecting a concentration of 0.5 mol/L KCl solution (2~3 mL) at the perioral membrane of the parent sea urchin. Nine females and three males, with a shell diameter of 5~6 cm and a body weight at 90~110 g, were used for this purpose. Then, they were placed in a breaker filled with seawater to let the gametes discharge. Mature sea urchins can release gametes within 5 min of injection. The eggs produced in one hour were collected and placed in a 20 L bucket and left standing for 10~15 min. We slowly poured off the upper seawater and repeated this 2–3 times to obtain high-quality eggs for artificial fertilization. A small number of eggs (10~50 eggs/mL) were collected in a 100 mL beaker and mixed with sperm. The appropriate sperm density was 5 sperm around each egg under a microscope. Next, adequate quality sperm was added to the eggs according to proportion. Five minutes after fertilization, the zygote can be transferred to the hatching barrel (800 L), and the water temperature should be controlled at 29.5 °C.

### 4.2. Larval Culture

The larvae were cultured following the general culturing methods of echinoderm larvae [68]. During the process of larval cultivation, seawater was filtered by a 0.01 μm filtration system, and temperature was maintained at 28.5~31 °C, salinity at 29.8~30.3, pH at 7.8~8.2, and dissolved oxygen at 5.9~6.4 mg/L. We fed *Chaetoceros muelleri* from the two-armed larvae twice a day, with a feeding amount controlled at 1 × 10^4^ cell/mL. The amount was gradually increased to 2 × 10^4^ cell/mL for four-armed larvae, 3 × 10^4^ cell/mL for six-armed larvae, and the density was controlled at 1/mL before six-armed larvae, then decreased to 0.5/mL at the eight-armed larvae. We added *Isochtysisi galbana* to the eight-armed larvae, with a quantity of a half of that of *C. muelleri*. The total feeding amount remained 4~5 × 10^4^ cell/mL for eight-armed larvae, and they were fed three times a day. Water was changed daily, with 1/3 of the total volume replaced each time.

After six days, most of the larvae developed to the eight-armed stage (the third stage described by Smith et al. [3]). We used 200 mesh silk screen (74 μm) to softly fish out these larvae and then observe their morphology under a microscope. Once confirmed as eight-armed larvae, about 3000 individuals were packed in foam tanks filled with seawater and transported to Genedenovo Company for single-cell RNA sequencing.

Without adding any substratum, when most larvae were settled at the bottom, the larvae in the upper layer of the water were fished out. Then, they were induced with 0.05 mmol/L KCl, and larvae that could settle within 30 min were treated as competent larvae. We packed about 3000 individuals according to the method of collecting eight-armed larvae.

We sucked out the juvenile from the bottom with a 5 mL rubber-tip dropper every two hours for observation. When most of the larvae finished metamorphosis, about 500 individuals would be transferred to a plastic bucket, and then observed and packed. A portion of the new juveniles (settled for one day) were transferred to cages and fed with gracilaria, laver, and sea lettuce. The water was changed once a week, with one-third of the water changed each time. After three days, the juveniles that survived and were able to feed were defined as competent juveniles. About 200 competent juveniles were collected for sequencing.

### 4.3. Scanning Electron Microscopy Images of Sea Urchins

The harvesting of fresh individuals to visualize the developmental stages during the metamorphosis content is performed. Subsequently, delicately wash the individuals with PBS (Servicebio, Wuhan, China, code G0002). Following the washing step, the individuals should be promptly fixed with electron microscopy fixative (Servicebio, code G1102) for 2 h at room temperature. After fixation, transfer the individuals to a 4 °C environment for preservation. Following fixation, rinse the individuals three times in 0.1 M PBS (pH 7.4), with each wash lasting for 15 min. Subsequently, immerse the individuals in 1% OsO4 solution (Ted Pella Inc, Redding, CA, USA) in 0.1 M PBS for 1–2 h at room temperature. After the OsO4 treatment, repeat the washing process with 0.1 M PBS three times, with each wash lasting for 15 min. The individuals should undergo dehydration using a series of ethanol solutions (from Sinaopharm Group Chemical Reagent Co. LTD, Shanghai, China, code 100092183) as outlined below: Immerse the individuals in 30% ethanol for 15 min. Subsequently, transfer them to 50% ethanol for another 15 min. Progress to 70% ethanol for a 15 min incubation. Continue the dehydration process by placing the individuals in 80% ethanol for 15 min. Follow with 90% ethanol for another 15 min. Transition to 95% ethanol for 15 min. Complete two successive immersions in 100% ethanol, each lasting for 15 min. Lastly, dehydrate the individuals in isoamyl acetate (Sinaopharm Group Chemical Reagent Co. LTD, code 10003128) for a final 15 min interval. Samples were dried with Critical Point Dryer (Quorum, East Sussex, UK, K850). To apply a conductive metal coating, specimens are affixed to metallic stubs using carbon stickers and then sputter coated with gold for a duration of 30 s. The elemental analysis of the target area was examined using a scanning electron microscope (Hitachi, Tokyo, Japan, SU8100).

### 4.4. Single-Cell RNA Sequencing

The dissociation of samples into individual cells was carried out as outlined by Paganos et al. [15]. In summary, larvae and juveniles were collected and the seawater was removed before resuspending them in calcium- and magnesium-free artificial seawater. Subsequently, the larvae were transferred to a dissociation buffer composed of 1 M glycine and 0.02 M EDTA in the same calcium- and magnesium-free seawater. They were then incubated on ice for 10 min, during which gentle mixing was performed by pipette aspiration every two minutes. Following this incubation period, the dissociation process was closely monitored.

We sequenced using a 10X Genomics single-cell capture system. First, we took some cell suspension and added AO/PI (acridine orange/propidium iodide) of equal volume. Then, we used Countess^®^ II Automated Cell Counter to count cells number and adjust the concentration of living cells to 1000~2000 cells/μL. The cDNA library was prepared according to the protocol of the Chromium Single Cell 3’ Reagent Kit (v3) (10X Genomics, Pleasanton, NJ, USA).

Next, the cDNA was labeled. Gel beads containing barcodes combined with the mixture of cells and enzymes, and then formed GEMs (gel beads-in-emulsions). Upon dissolution of the gel bead in a GEM, primers containing (i) an Illumina^®^ R1 sequence (read 1 sequencing primer), (ii) a 16 nt 10x Barcode, (iii) a 10 nt Unique Molecular Identifier (UMI), and (iv) a poly-dT primer sequence were released and mixed with cell lysate and Master Mix. Barcoded, full-length cDNAs were then reverse-transcribed from poly-adenylated mRNA. The products of all GEMs were mixed to construct a standard sequencing library. Then, the cDNA was digested into fragments of 200~300 bp, and the DNA library was obtained by being end repaired, with added poly A, P5, P7, sample index, and PCR amplification. Finally, the PE150 sequencing platform was used for high-throughput sequencing.

### 4.5. Data Quality Control and Gene Expression Quantification

The data quality control was performed using Cell Ranger (version 3.1.0) (http://support.10xgenomics.com/single-cell/software/overview/welcome, accessed on 27 January 2022), and then all reads were mapped to the full-length transcriptome of *H. crassispina* [69] (NCBI accession number PRJNA767828, SRR16149279). After the correction and statistics of UMI (unique molecular identifier), we identified and distinguished the cells and non-cells in the data. Further, DoubletFinder (version 2.0) [70] was implemented to calculate the probability value that the GEM was multicellular, and then it was filtered.

Then, the Seurat version 3.1.1 [71] was adopted to filter cells according to the following criteria: (i) the gene counts in each cell is 500~4000; (ii) the number of UMI is less than 8000; (iii) the mitochondrial gene proportion < 10%. The gene expression was normalized as follows: a gene expression level = log (1 + (UMI A/UMI Total) × 10,000).

After removing low-quality cells, data consolidation and batch effect correction were carried out using Harmony [72]. We used Seurat, which utilized canonical correlation analysis and mutual nearest neighbor analysis, to aggregate all samples [73]. Two thousand highly variable genes were selected in each sample based on a variance-stabilizing transformation. Anchors between individual data were identified and correction vectors were calculated to generate an integrated expression matrix, which was used for subsequent clustering. Then, we distributed cells to clusters.

The integrated expression matrix was then scaled and performed on principal component analysis for dimensional reduction. Then, we implemented a resampling test inspired by the jackStraw procedure. We randomly permuted a subset of the data (1% by default) and reran PCA, constructing a ‘null distribution’ of gene scores, and repeated this procedure. We identified ‘significant’ PCs as those who had a strong enrichment of low *p*-value genes for downstream clustering and dimensional reduction [71].

Seurat implements a graph-based clustering approach. Distances between the cells were calculated based on previously identified PCs. Briefly, Seurat embedded cells in a shared-nearest neighbor (SNN) graph, with edges drawn between cells via similar gene expression patterns. To partition this graph into highly interconnected quasi-cliques or communities, we first constructed the SNN graph based on the Euclidean distance in PCA space and refined the edge weights between any two cells based on the shared overlap in their local neighborhoods (Jaccard distance). We then applied modularity optimization techniques—SLM [74] to iteratively group cells together, with the goal of optimizing the standard modularity function.

The t-SNE (t-Distributed Stochastic Neighbor Embedding) [75] nonlinear clustering method was used to visualize the clusters.

### 4.6. Bioinformatics Analysis

We used a likelihood ratio test to find the DEGs (differential expression genes) between one cluster and other clusters. The DEG was determined according to the following criteria: (i) *p*-value < 0.01; (ii) log2FC ≥ 0.36; (iii) the cell percentage with this gene detected in a cluster >25%. GO [76] analysis was also performed to analyze the functions of DEGs.

To find out biologically significant modules, WGCNA (version 1.47) [77] analysis was performed. Next, module eigengenes were used to calculate the correlation coefficient with samples. For genes in each module, GO enrichment analyses were conducted to analyze the biological functions of modules. Then, to find the hub genes of each cluster, we selected the first 200 interactions in each module according to weight value and used the MCC topology analysis method of plug-in CytoHubba in Cytoscape [78] to display the top ten hub genes.

Finally, pseudotime analysis was analyzed using PAGA [79] and Monocle 2 [80]. The PAGA graph was made using the preprocessed Seurat object. For this, the Scanpy (v1.6.0) function scanpy.pp.neighbors was run using the PCA embeddings calculated by Seurat. Then, scanpy.tl.paga was run using Seurat clusters as groups, and finally, the plot was generated using the function sc.pl.paga_compare. PAGA achieves consistent and topology-preserving embeddings by initializing an embedding of a fine-grained graph using the coordinates of a coarse-grained graph. PAGA-initialized ForceAtlas2 mapped cells in an easily interpretable manner. After assigning the starting cell, the software automatically calculated the pseudotime value of each cell by referring to the DPT algorithm.

Single-cell trajectory was analyzed using the matrix of cells and gene expressions by Monocle (Version 2.10.1). Monocle reduced the space down to one with two dimensions and ordered the cells (sigma = 0.001, lambda = NULL, param.gamma = 10, tol = 0.001) [81]. Once the cells were ordered, we could visualize the trajectory in the reduced dimensional space. The trajectory has a tree-like structure, including tips and branches.

All figures were illustrated using ggplot2 in R [82] and the Omicsmart platform (https://www.omicsmart.com).

### 4.7. Quantitative Reverse Transcription–Polymerase Chain Reaction (qRT-PCR) Analysis

A total of 23 genes from six cell populations (Supplementary File S3) were selected to test if the transcripts were indeed detected at relatively similar levels at these four stages, and we utilized qRT-PCR to measure the transcripts’ abundance in dissociated samples (whole larvae and juveniles) samples. Additionally, the expression patterns of genes in the early stage (four-armed and six-armed larvae) were also evaluated.

Total RNA extraction from each sample was performed following the manufacturer’s protocol using the Eastep™ Super Total RNA Extraction Kit (Promega, Shanghai, China). Subsequently, first-strand cDNA was synthesized with random hexamers utilizing M-MLV reverse transcriptase (Promega, Shanghai, China) as per the manufacturer’s guidelines. The expression levels of the candidate genes were evaluated through qRT-PCR. The total reaction volume was 10 μL, comprising GoTaq^®^ qPCR Master Mix (5 μL), forward primer (10 mM) (0.25 μL), reverse primer (10 mM) (0.25 μL), and cDNA template (4.5 μL). The reactions were conducted at 95 °C for 2 min, followed by 40 cycles of 95 °C for 15 s and 60 °C for 1 min. All primer sequences are listed in Supplementary file S3, with *GAPDH* serving as the internal reference gene. Data analysis was performed using ANOVA in SPSS Statistics 22, considering variances statistically significant for *p* < 0.05. Subsequently, the data were graphically presented using Origin software (version 2021).

## 5. Conclusions

In summary, our study has reported the cell states of *H. crassispina* using scRNA-seq, identified 22 clusters, including nerve cells, skeletogenic cells, immune cells, digestive cells, germ cells, and muscle cells. Four significant outcomes of the analysis are as follows: (1) the larval peripheral nerves were discarded during metamorphosis; (2) revealed that the cell abundance of spicules decreases during metamorphosis; (3) explored the characteristic of larval and adult pigment cells; (4) cellulase is critical for competent juveniles. These data may deepen our understanding on an important and vastly under-appreciated topic: what are the molecular mechanisms underlying the transition of marine larvae from the plankton to the seafloor? To date, many key issues related to this topic remain unclear, and further research may focus on the following questions: How do larvae deploy behaviors to increase the likelihood of settling in the right time and place? How does a single genome produce two totally different forms in different habitats? And why (and how) do some taxa settle ‘spontaneously’ on nearly any substratum whereas other taxa maintain extreme specificity? Yet, despite its commonality and import, we still lack a basic mechanistic/molecular understanding of this process in any taxon and have a long way to go before we can really answer any of these aforementioned key questions.

## Data Availability

The single-cell RNA-seq data generated in this study have been deposited in the Genome Sequence Archive in BIG Data Center (http://bigd.big.ac.cn/), Beijing Institute of Genomics (BIG), Chinese Academy of Sciences, under the accession number: CRA009256.

## Supplementary Materials

The supporting information can be downloaded at: https://www.mdpi.com/article/10.3390/ijms26031059/s1.
