# Supplementary material for: The Cell States of Sea Urchin During Metamorphosis Revealed by Single-Cell RNA Sequencing
Source: Int J Mol Sci. 2025 Jan 26;26(3):1059. doi: 10.3390/ijms26031059 (PMC11817407; doi:10.3390/ijms26031059)
Supplement: Supplementary file 1 [file ijms-26-01059-s001.zip › Supplementary File S3. The primers and qPCR results .pdf]

Table 1. The gene information for qPCR

Cell states	ID	Gene	Primers	Product length (bp)
NC	Isoform0004998	*An*	F: CATGCCAGTCAGCTAGGACA<br>R: ATCCGATGGAGATCTGTTGC	109
	Isoform0007334	*F-salmfa*	F: GTCTTCACCGCCAATGATCT<br>R: CTTGGAGGCTGTCGTAGAGG	143
	Isoform0021121	*Tubb4B*	F: GCTGAGGAGGAGGGAGAGTT<br>R: TCAAATCTTTGGTGGTGCAA	140
	Isoform0007238	*Lim1*	F: ATGGACCATCACATCCCTGT<br>R: AAACATGTCCCCGTTAGCTG	147
SC	Isoform0000494	*Slc6a5*	F: CACCATCATGGAGACTGTGG<br>R: CATGATAGAGCAGGCGATCA	100
	Isoform0000260	*Slc4a10*	F: TCTCCGCCATGTTAAGCTCT<br>R: GGAAGACGATGGATGCCTTA	107
	Isoform0017753	*SM30A*	F: CTTCGATGCTGATTTGCTGA<br>R: GCATGTTTGTCGTTGTACCG	143
	Isoform0022945	*SM30*	F: ACAATTTCGGGAACCCTTTC<br>R: GCGGTAGTCACAGGACCATT	118
	Isoform0000040	*Col3A1*	F: AATTGCATCCCTCATCGAAC<br>R: GCATCCTTGACTGAGCCTTC	135
	Isoform0025783	*SM32*	F: GGATCGGGACCAGTAATGAA<br>R: GTCCTGGAGGGATGTTCTGA	127
PC	Isoform0018216	*Fmo6l*	F: AAGGCGATCGCATCATTTAC<br>R: ACTCGGGTGTCAGGAAACTG	121
	Isoform0025024	*FMO6L*	F: CTGGCCAAATCCTTCACAGT<br>R: TATGGGGTGACAGGTCCAAT	120
	Isoform0000054	*A2ml1*	F: CCATCATACGAGGGGAGAGA<br>R: GTTGCCACACACTTCAATGG	149
	Isoform0000245	*Dmbt1*	F: CTTAGCGGAAGGACGAGTTG<br>R: TTCACCAAGGGAGAGCATTC	115
	Isoform0002838	*Fmo2*	F: GTTCACATTCTGCGGGAGAT<br>R: GAAGTTGATGCGCTTGTTGA	149
	Isoform0017860	*Hox-D9a*	F: CATGTACCTGACGCGAGAGA<br>R: CATCTTCATCCGACGGTTCT	100
DC	Isoform0018526	*Cellulase*	F: GTCGGAGGTCCTGACGAATA<br>R: TCGTGATGAGTTGCTTGAGG	127
	Isoform0000432	*Endo16*	F: TCATTGCTGCGAGAAAGATG<br>R: ATCGTTGATGGGGTCTTCTG	148
	Isoform0003497	*Nrf-6*	F: TGGCTCTTATTGGCTCCATC<br>R: GGTCGTTGTAAAGCGGTGAT	111

GC	Isoform0009932	*H2A.V*	F: TCTGGAAAGGCAAAGGCTAA<br>R: GGCTCGTCGTTCTGTTCTTC	103
	Isoform0002291	*Gmnn*	F: GGCAGAGACACTGATCACGA<br>R: TCATCCTTGGGCTCATCTTC	143
MC	Isoform0018055	*Actin*	F: CCGAGCGTGGTTACTCTTTC<br>R: ACTTCTCGAGGGAGGAGGAG	135
	Isoform0022372	*Mp20*	F: ACAACGCGGAAACTGTAACC<br>R: CATCCTTGGACCCAAACAAT	112
Internal reference	Isoform0011283	*GAPDH*	F: ATGCCAAATCCAGCATTTTC<br>R: ACAACGCGGAAACTGTAACC	109

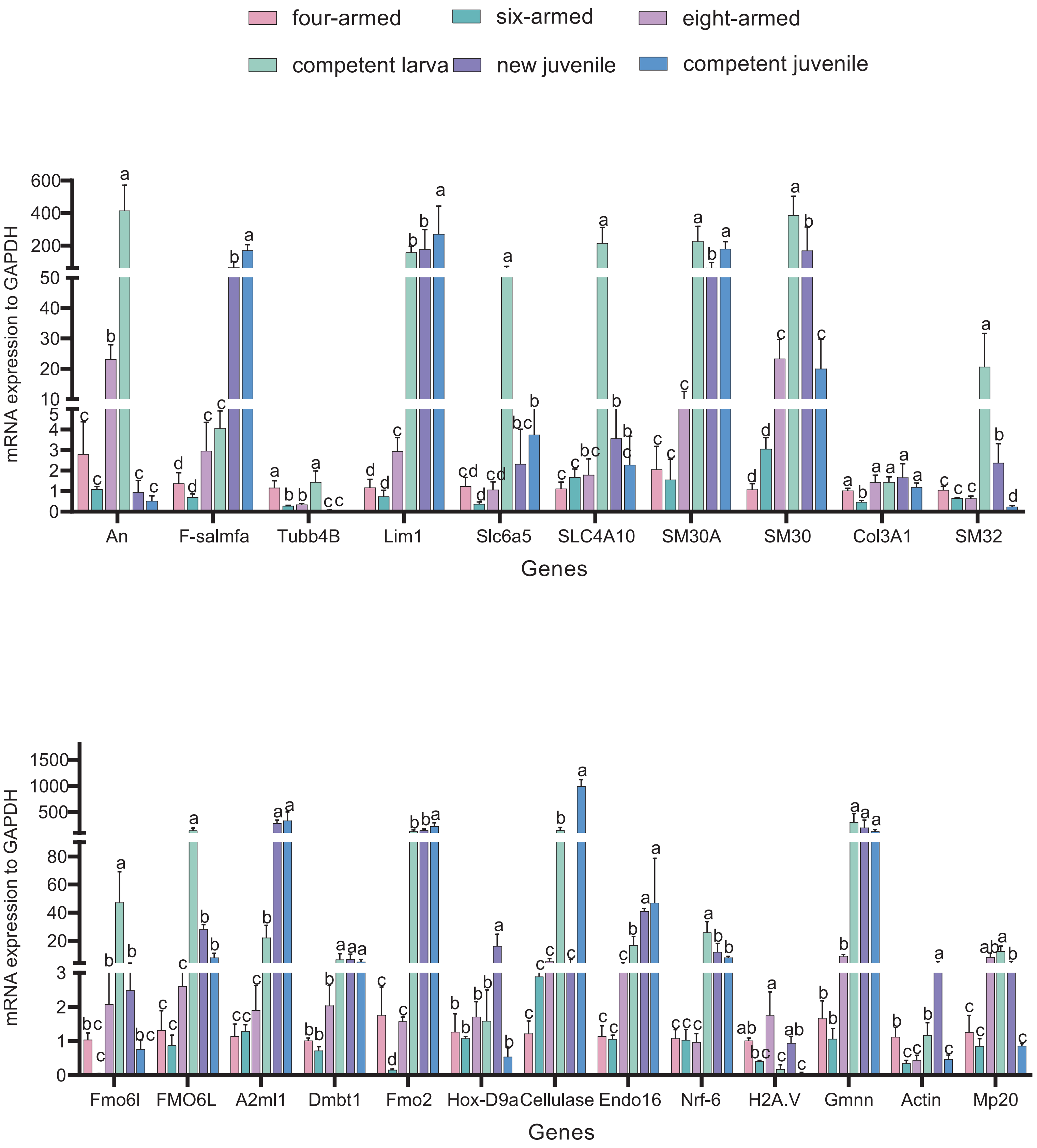

Figure 1. The qPCR results. Different letters representing significant differences between different developmental stages within the same gene
