# Supplementary material for: The Cell States of Sea Urchin During Metamorphosis Revealed by Single-Cell RNA Sequencing
Source: Int J Mol Sci. 2025 Jan 26;26(3):1059. doi: 10.3390/ijms26031059 (PMC11817407; doi:10.3390/ijms26031059)
Supplement: Supplementary file 1 [file ijms-26-01059-s001.zip › Supplementary File S4. WGCNA analysis.pdf]

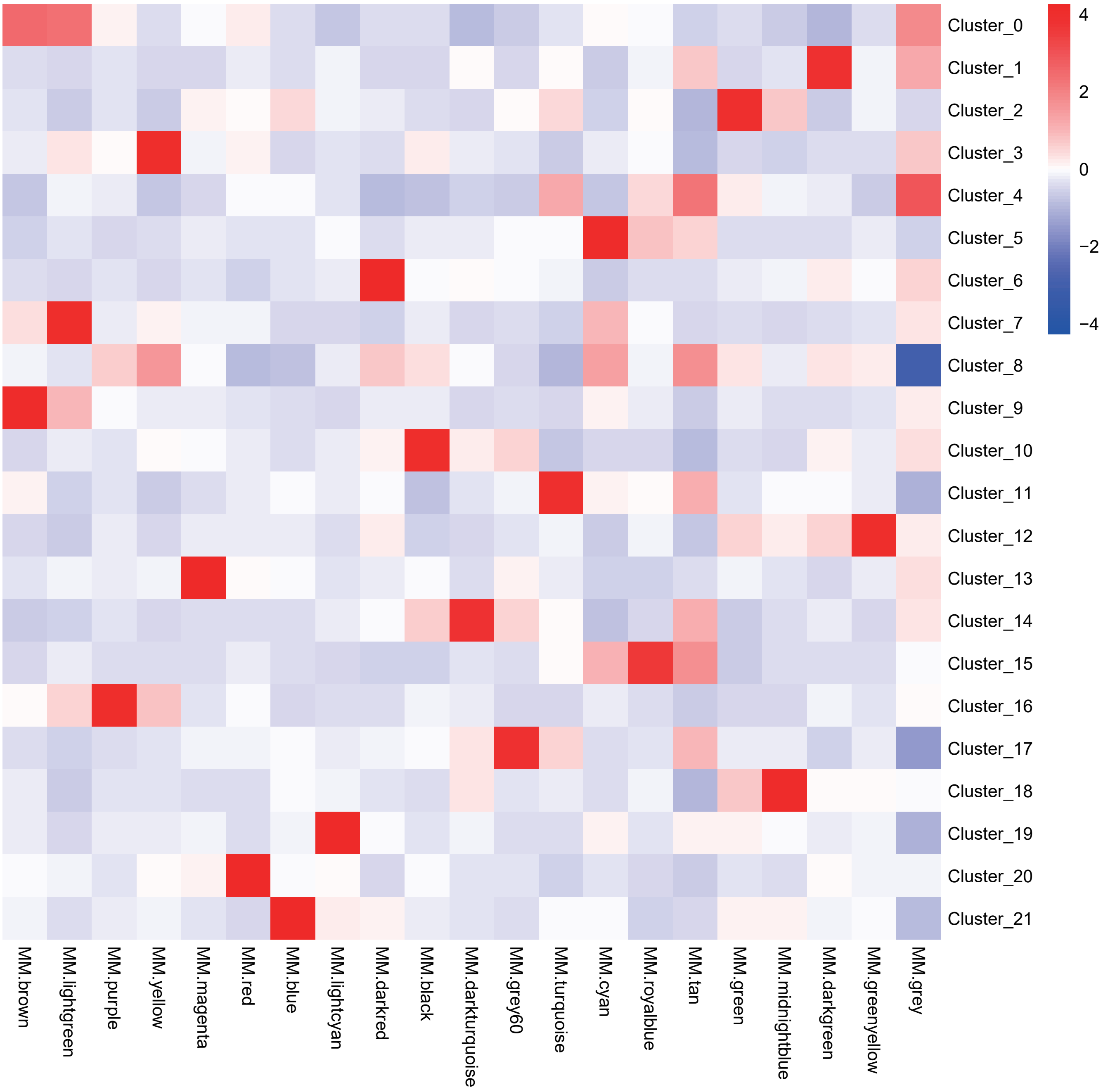

Supplementary Fig. 1. WGCNA analysis of 22 clusters identified 21 modules named in different colors.

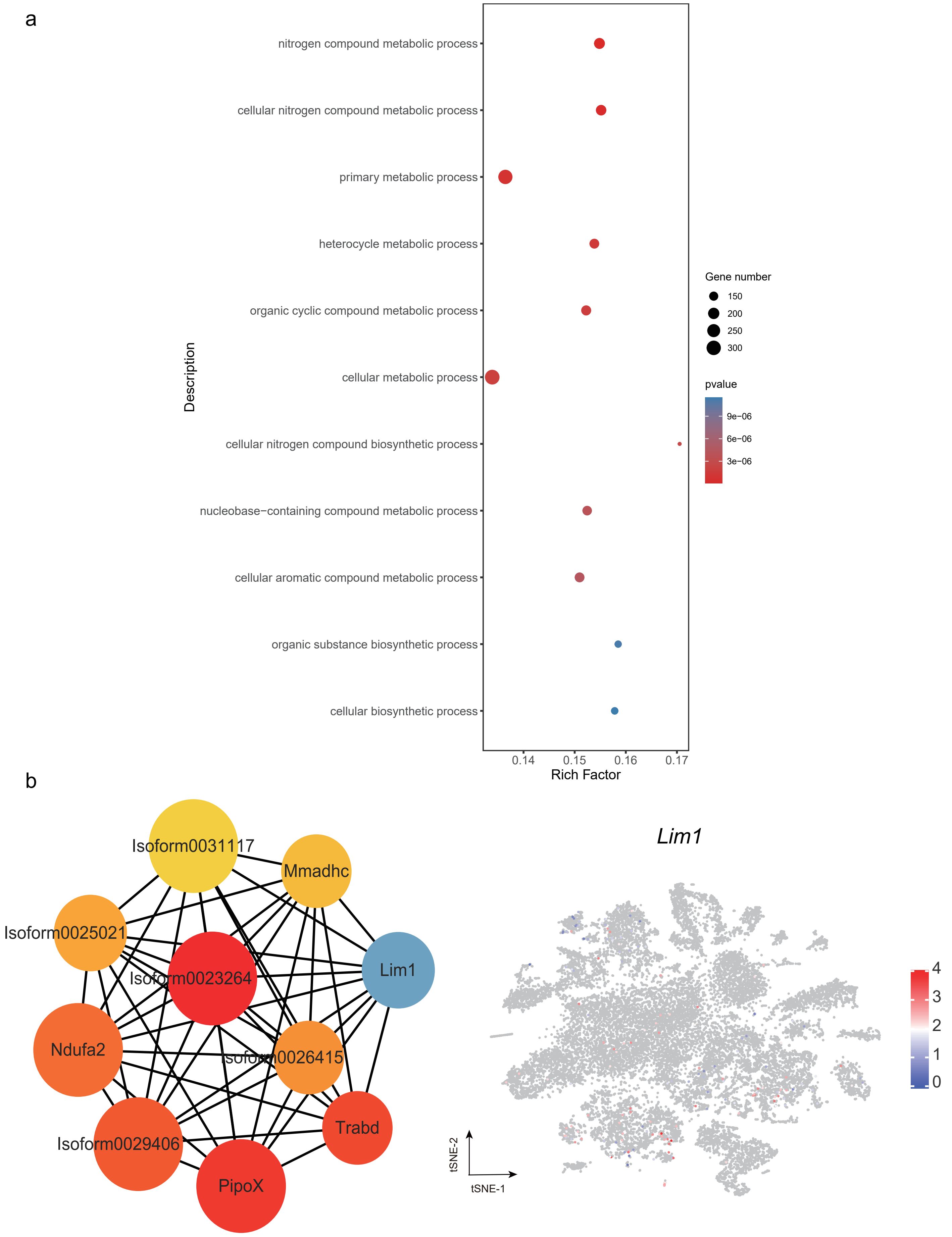

Supplementary Fig. 2. WGCNA analysis of the C21 in the NC population. (a) The GO terms show the main biological processes of the blue module that is related to C21. (b) The top 10 hub genes in the blue module, the transcript factor is labeled with blue color (left); t-SNE plot shows the distribution of transcription factor Lim1 (right).

a

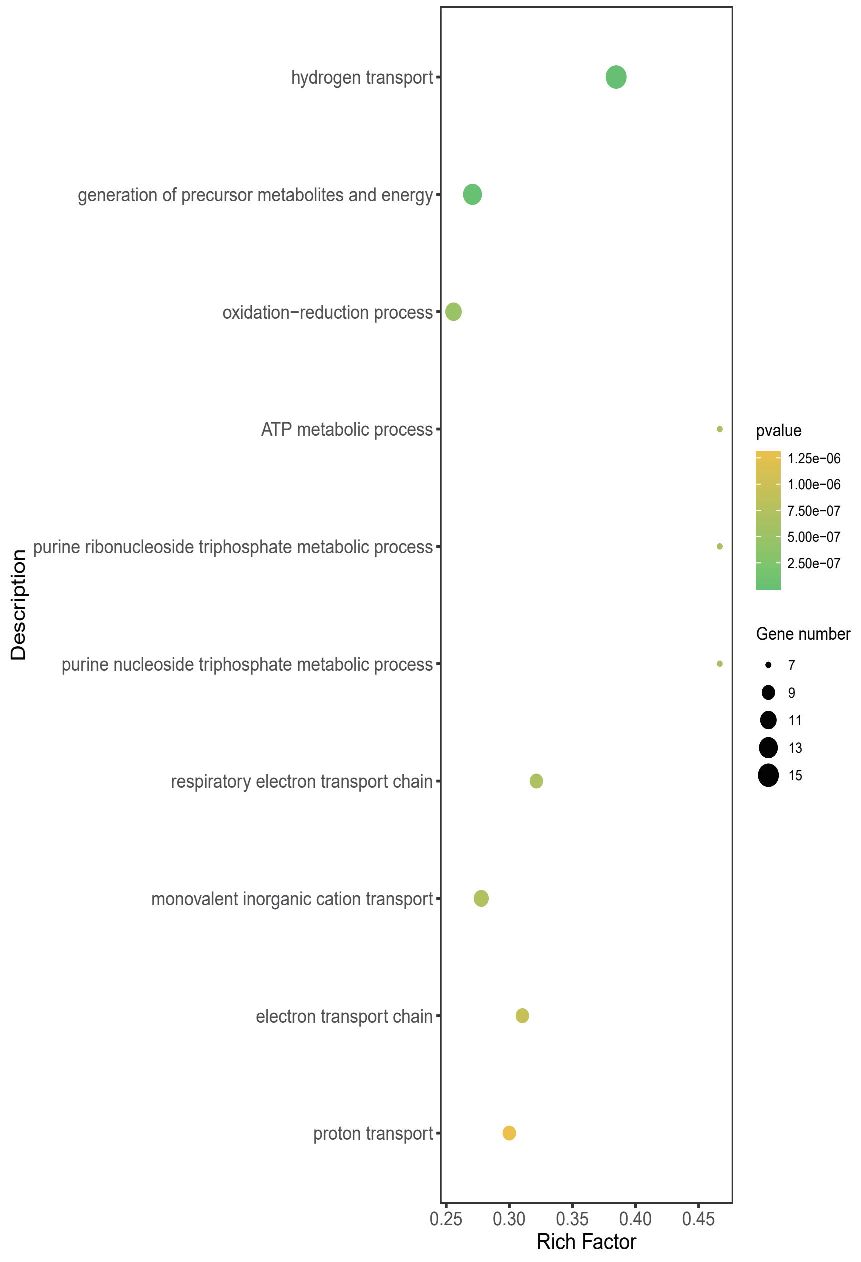

b

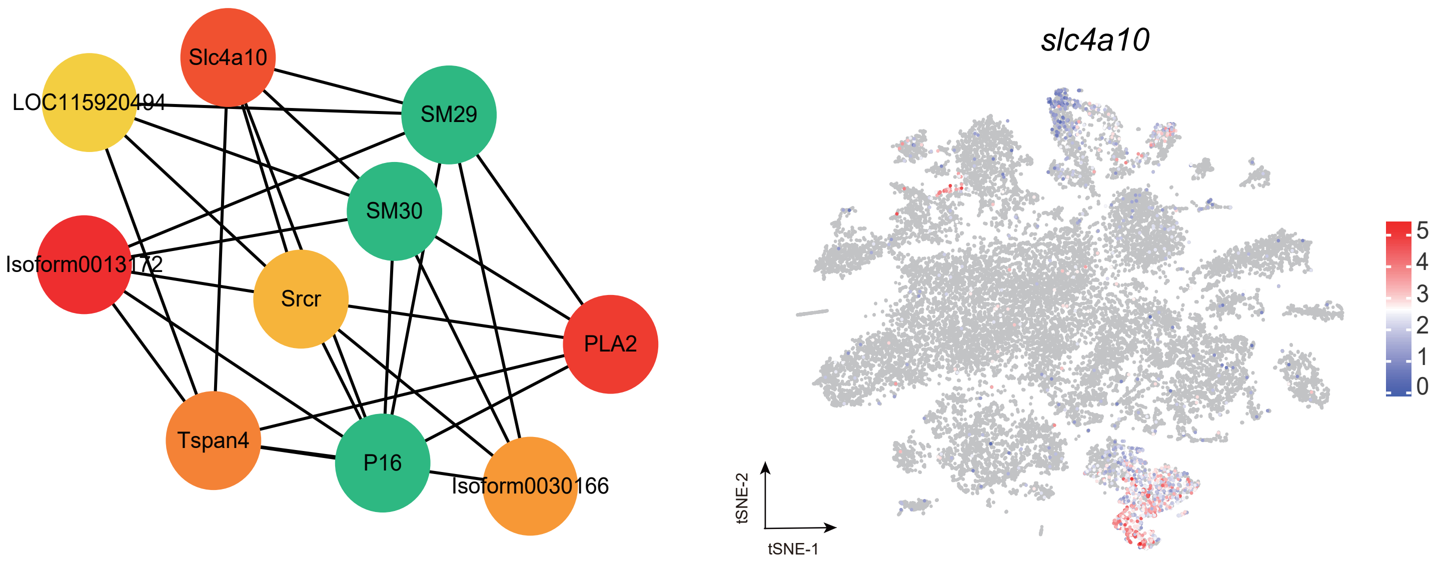

Supplementary Fig. 3. WGCNA analysis was performed in the C6 of the SC population.
(a) The bubble chart shows the top 10 biological processes of the darkred module that is associated with C6. (b) The top 10 hub genes in the darkred module, the marker genes used in this study are labelled with green color (left); t-SNE plot shows the distribution of slc4a10 (right).

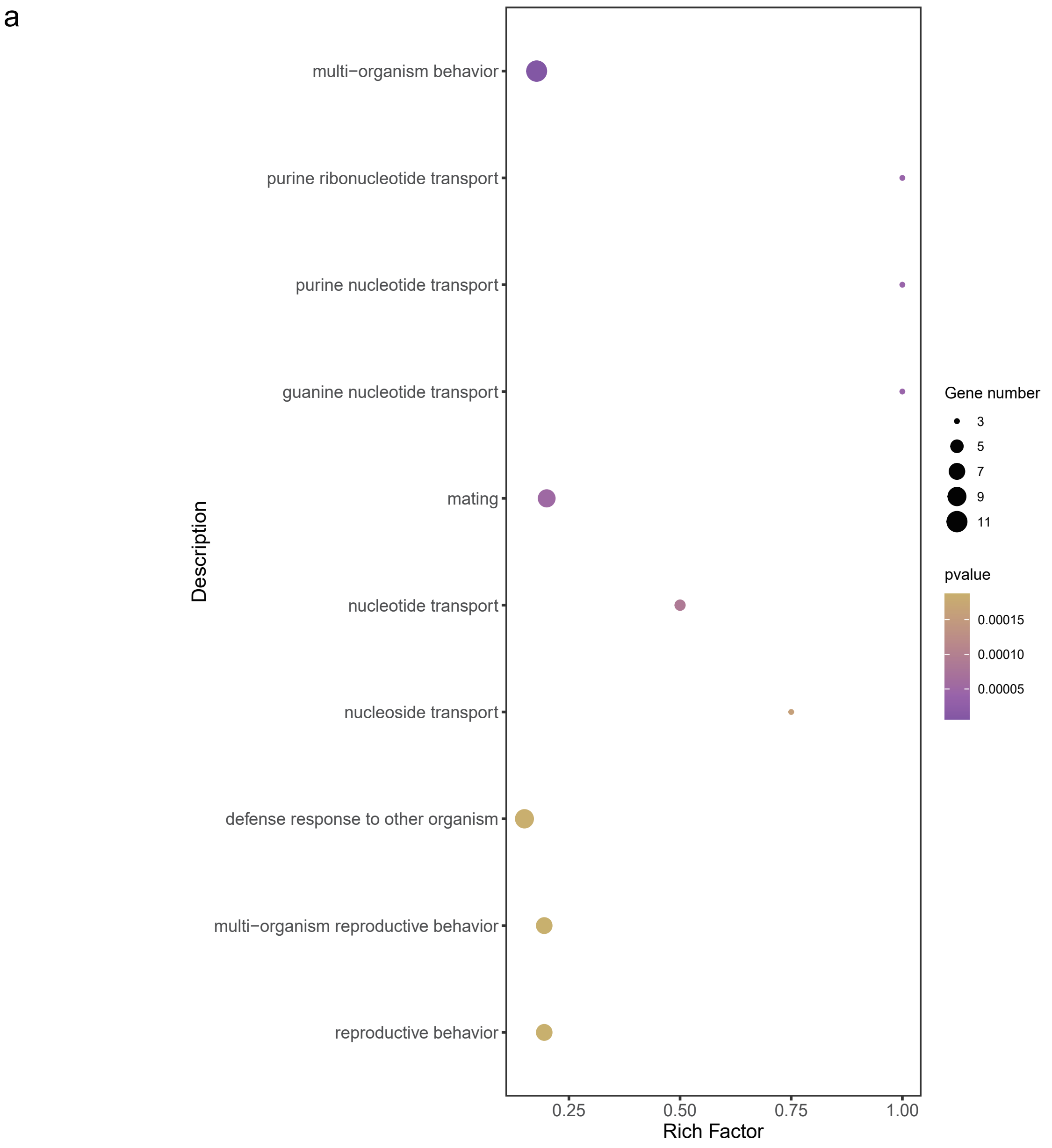

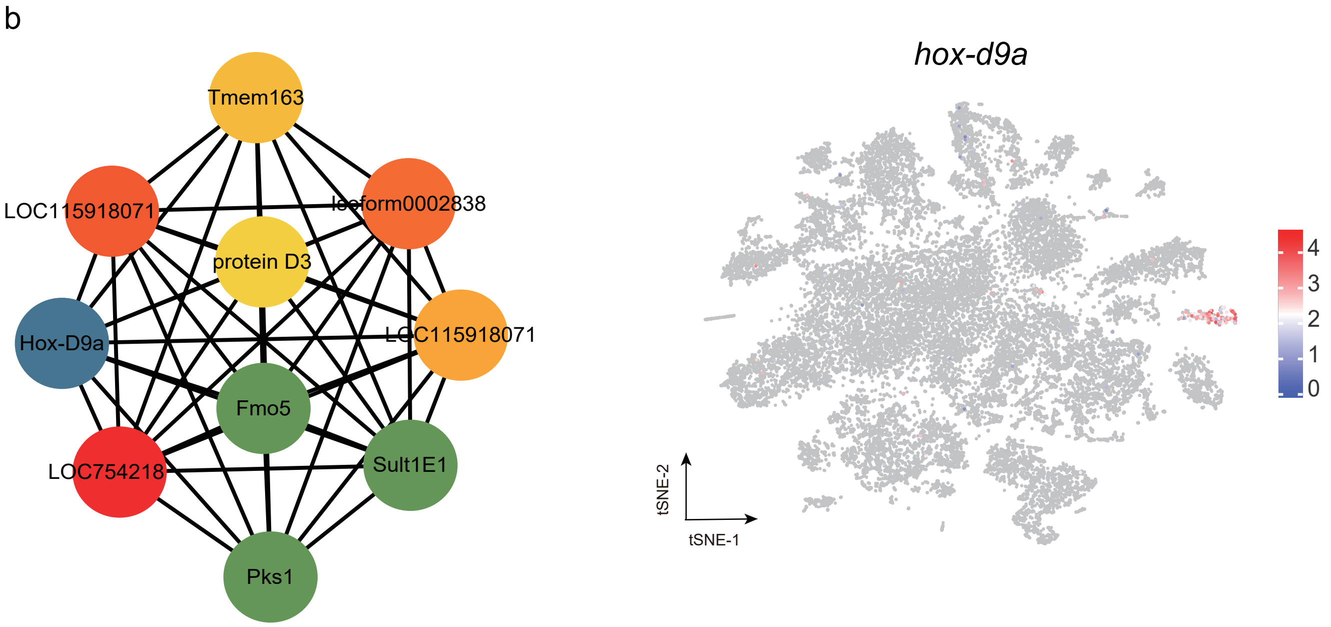

Supplementary Fig. 4. The gene co-expression network of PC was revealed by WGCNA analysis. (a) The bubble chart depicts the top 10 biological processes associated with the magenta module linked to C13. (b) The top 10 hub genes within the magenta module, including the marker genes and the transcription factor, are labeled with dark green and blue, respectively (left); the t-SNE plot delineates the distribution of hox-d9a (right).

a

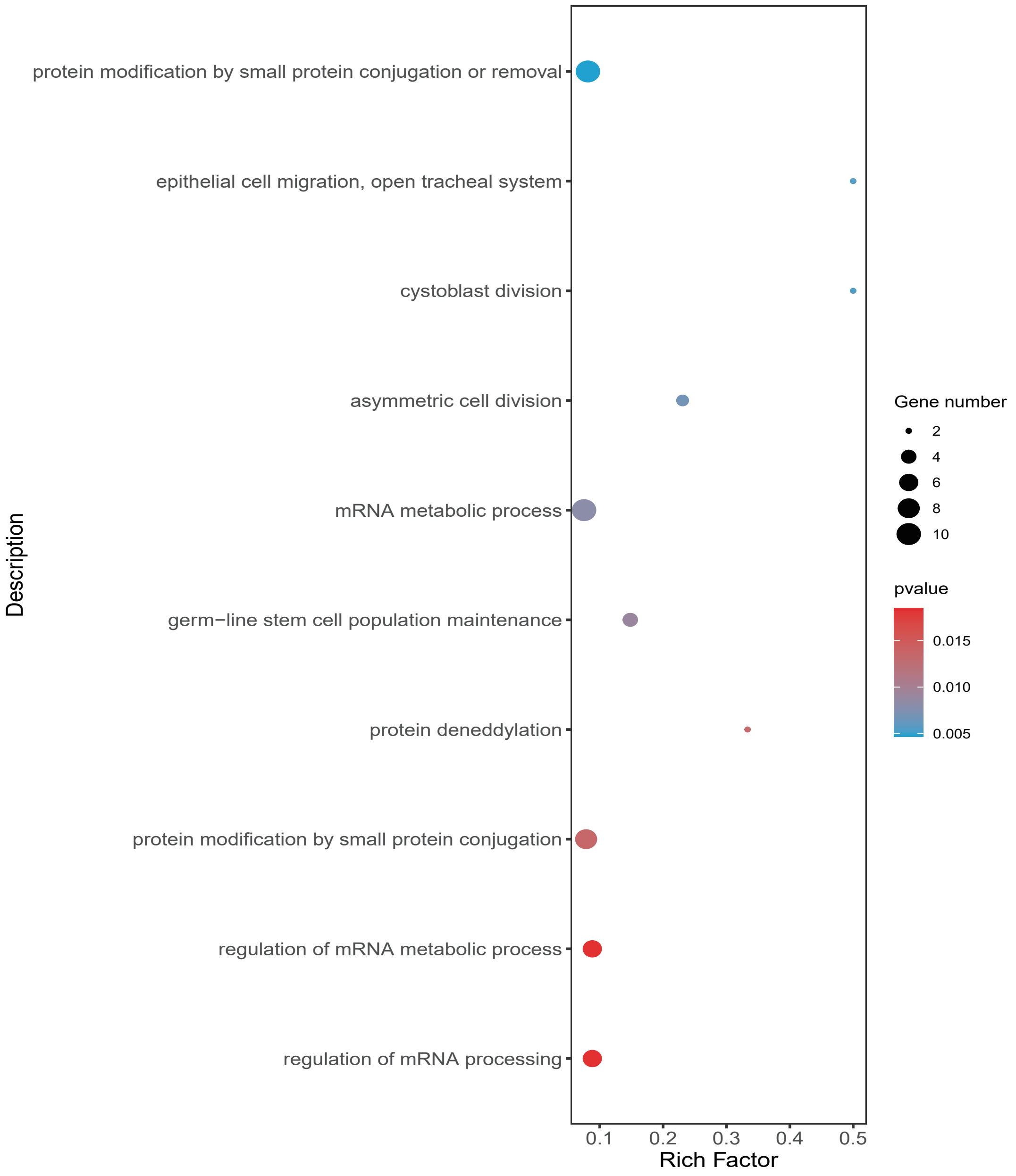

b

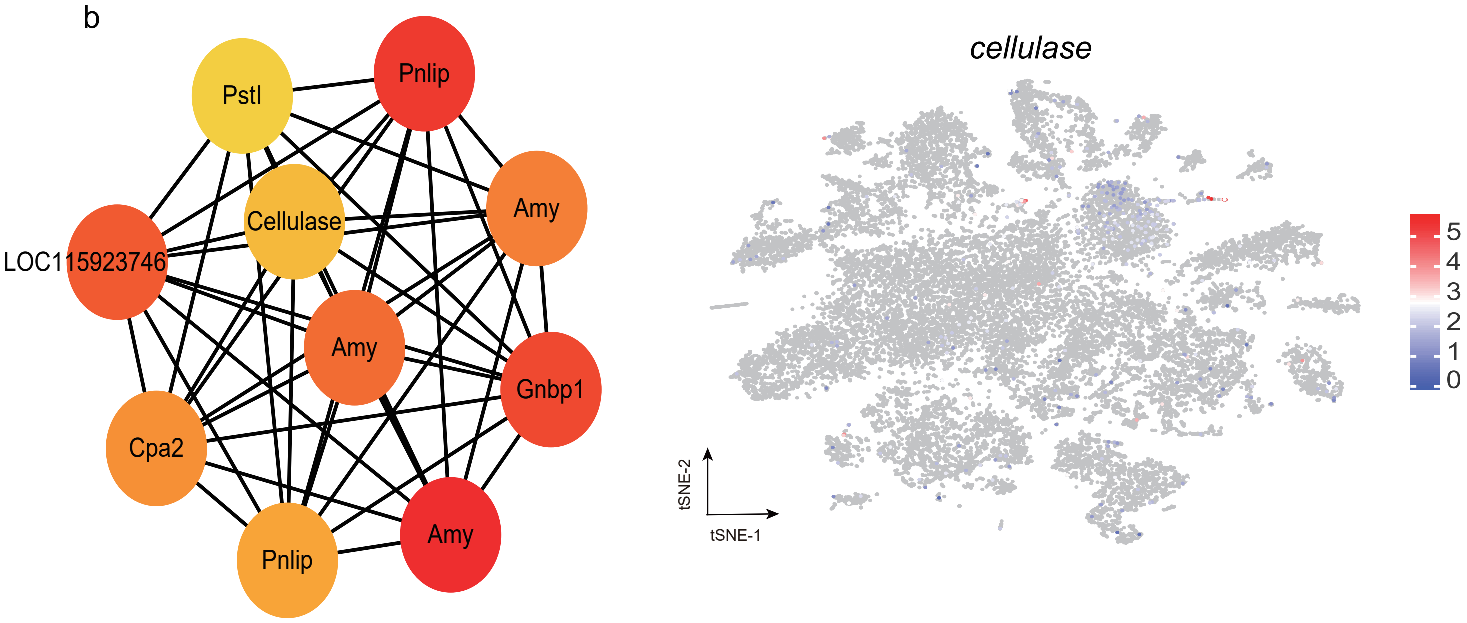

Supplementary Fig. 5. C16 of the DC population were analyzed by WGCNA. (a) The bubble chart shows the top 10 biological processes of the purple module involved with C16. (b) The top 10 hub genes in the purple module (left); the t-SNE plot shows the distribution of cellulase (right).
