# Supplementary figures and images for: The Cell States of Sea Urchin During Metamorphosis Revealed by Single-Cell RNA Sequencing
Source: Int J Mol Sci. 2025 Jan 26;26(3):1059. doi: 10.3390/ijms26031059 (PMC11817407; doi:10.3390/ijms26031059)

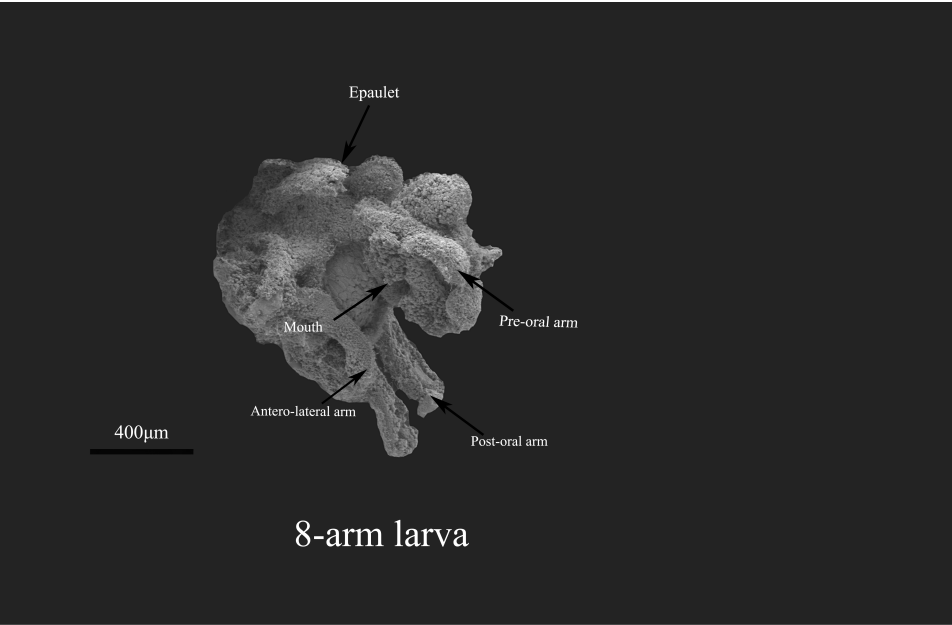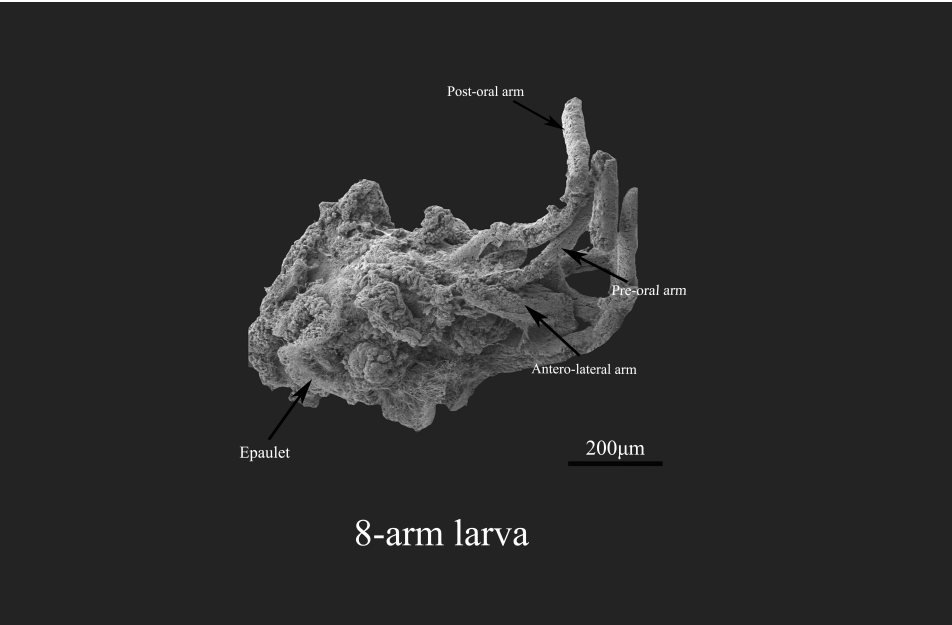

Eight-armed larva

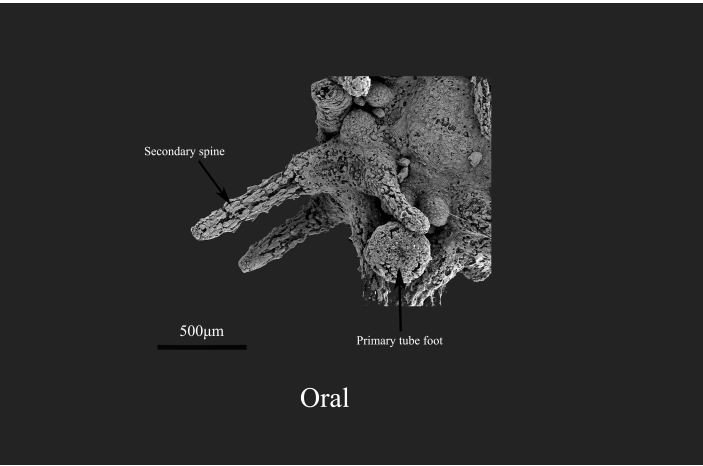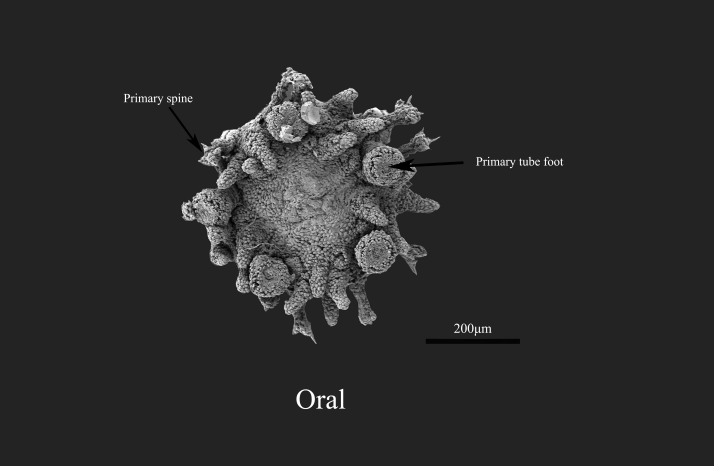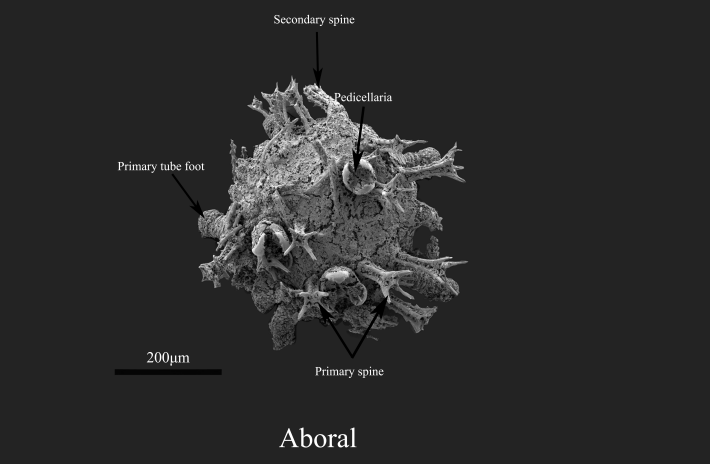

New juvenile

Supplement: Supplementary file 1 [file ijms-26-01059-s001.zip › Supplementary File S1. The scanning electron microscopy images of sea urchins.pdf]

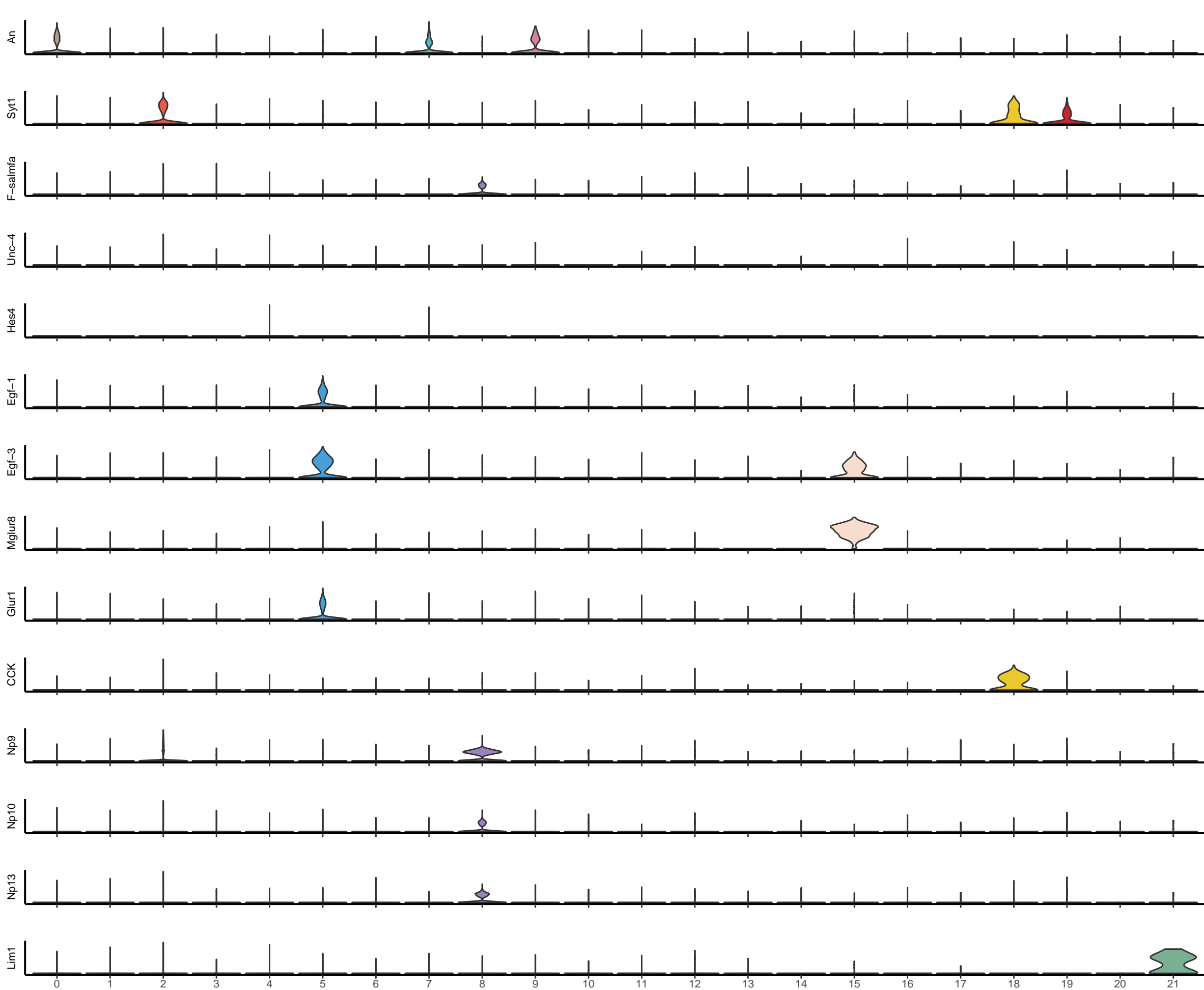

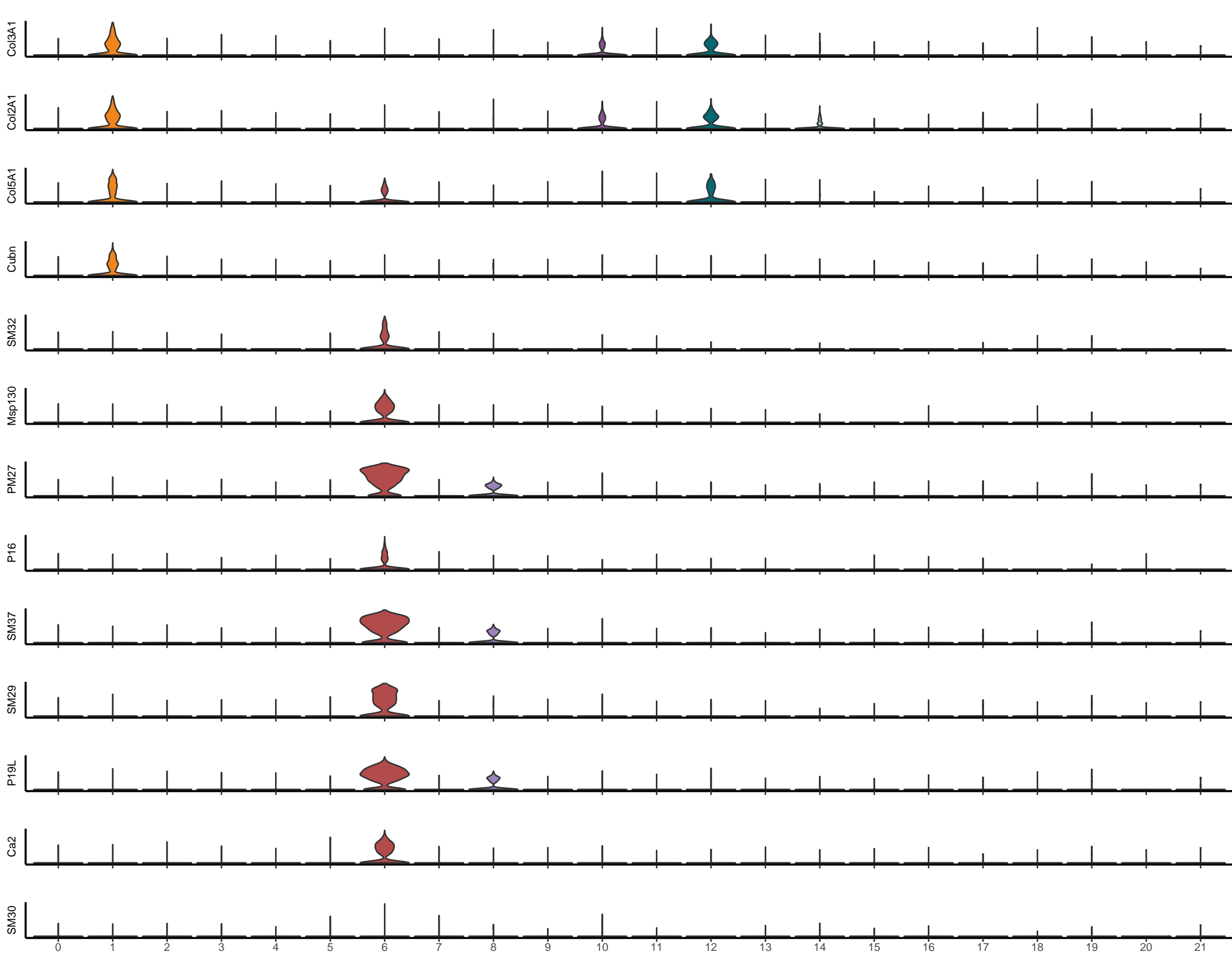

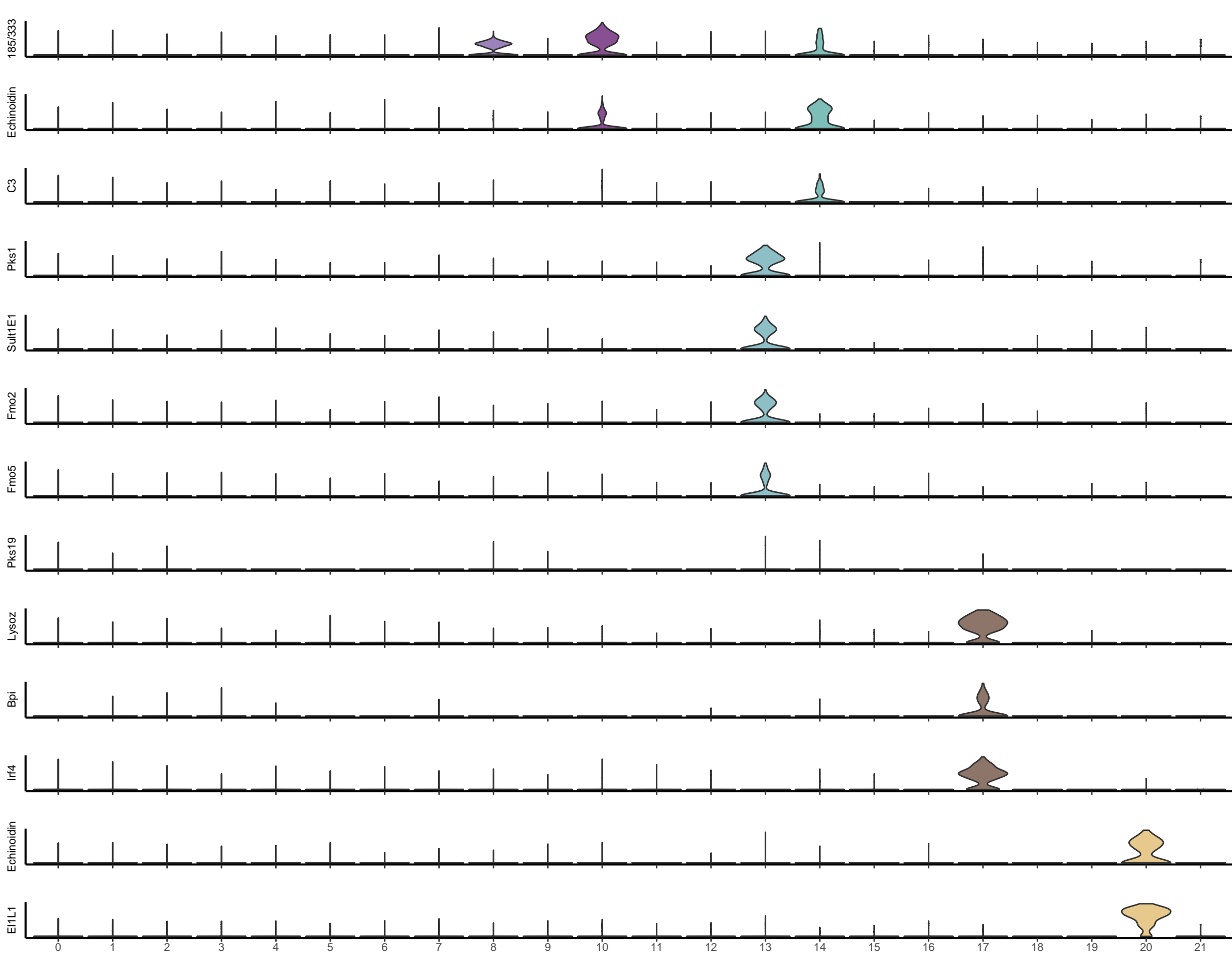

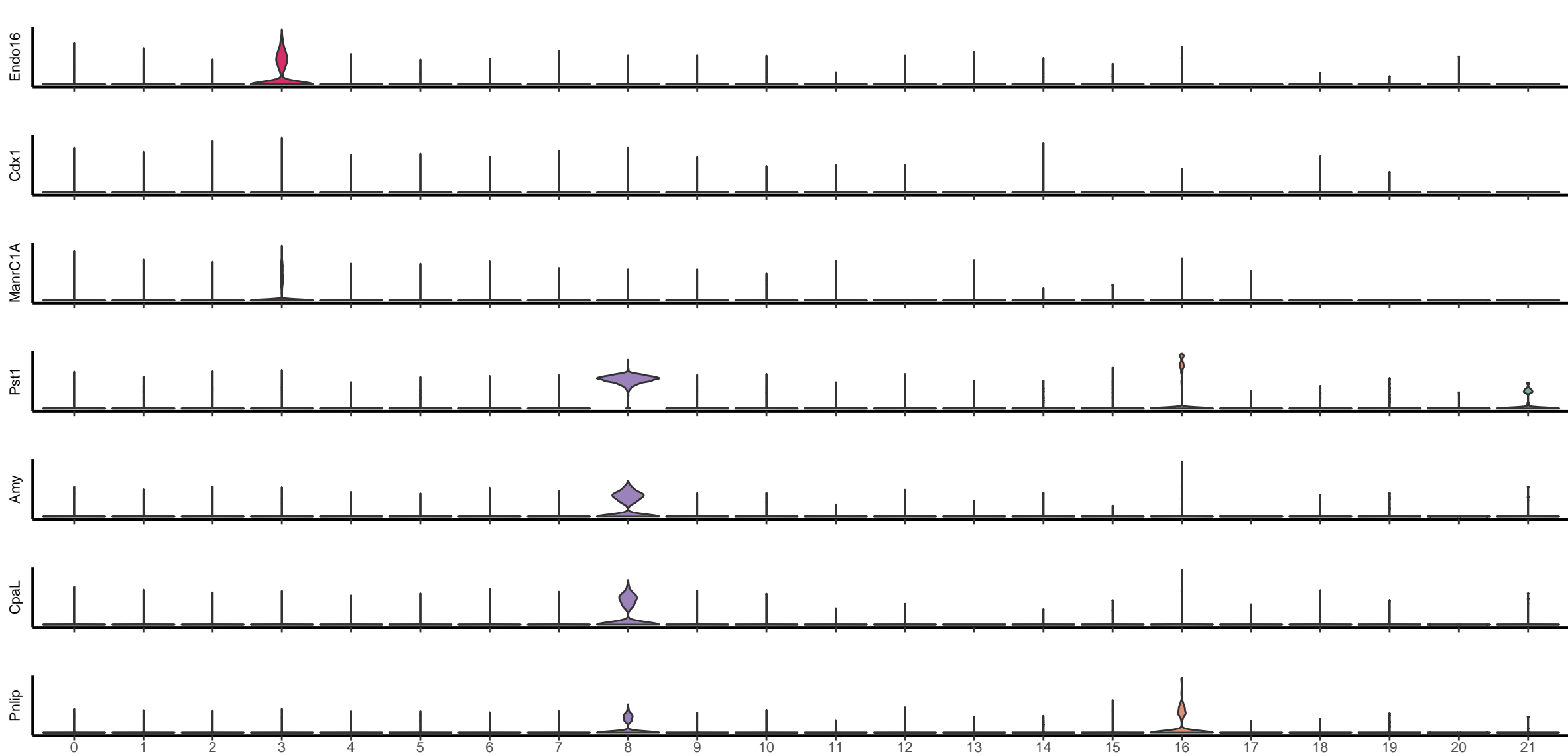

Supplement: Supplementary file 1 [file ijms-26-01059-s001.zip › Supplementary File S2. The marker genes used to identify the cell populations .pdf]

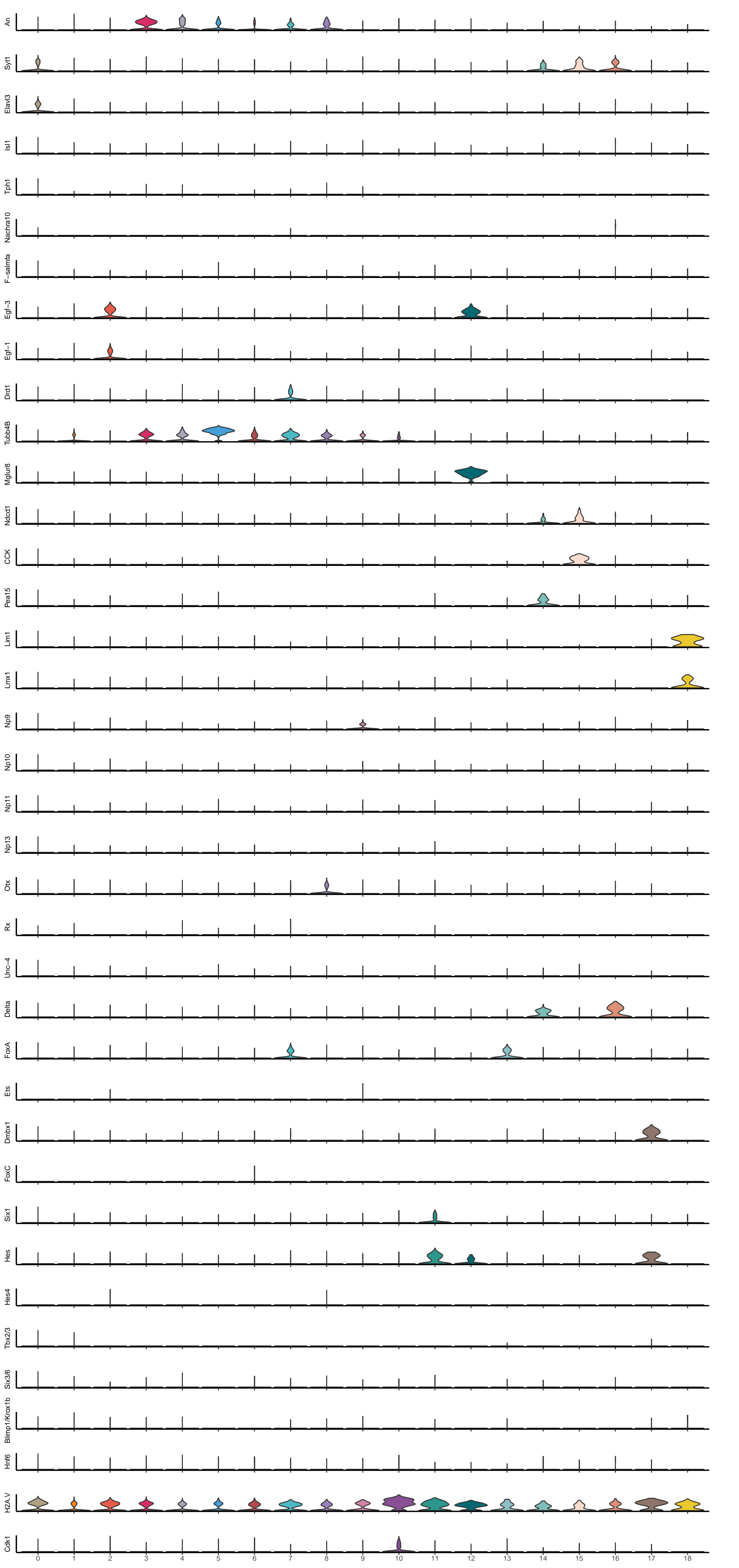

Supplement: Supplementary file 1 [file ijms-26-01059-s001.zip › Supplementary File S5. The expression patterns of marker genes in the NC subsets.pdf]

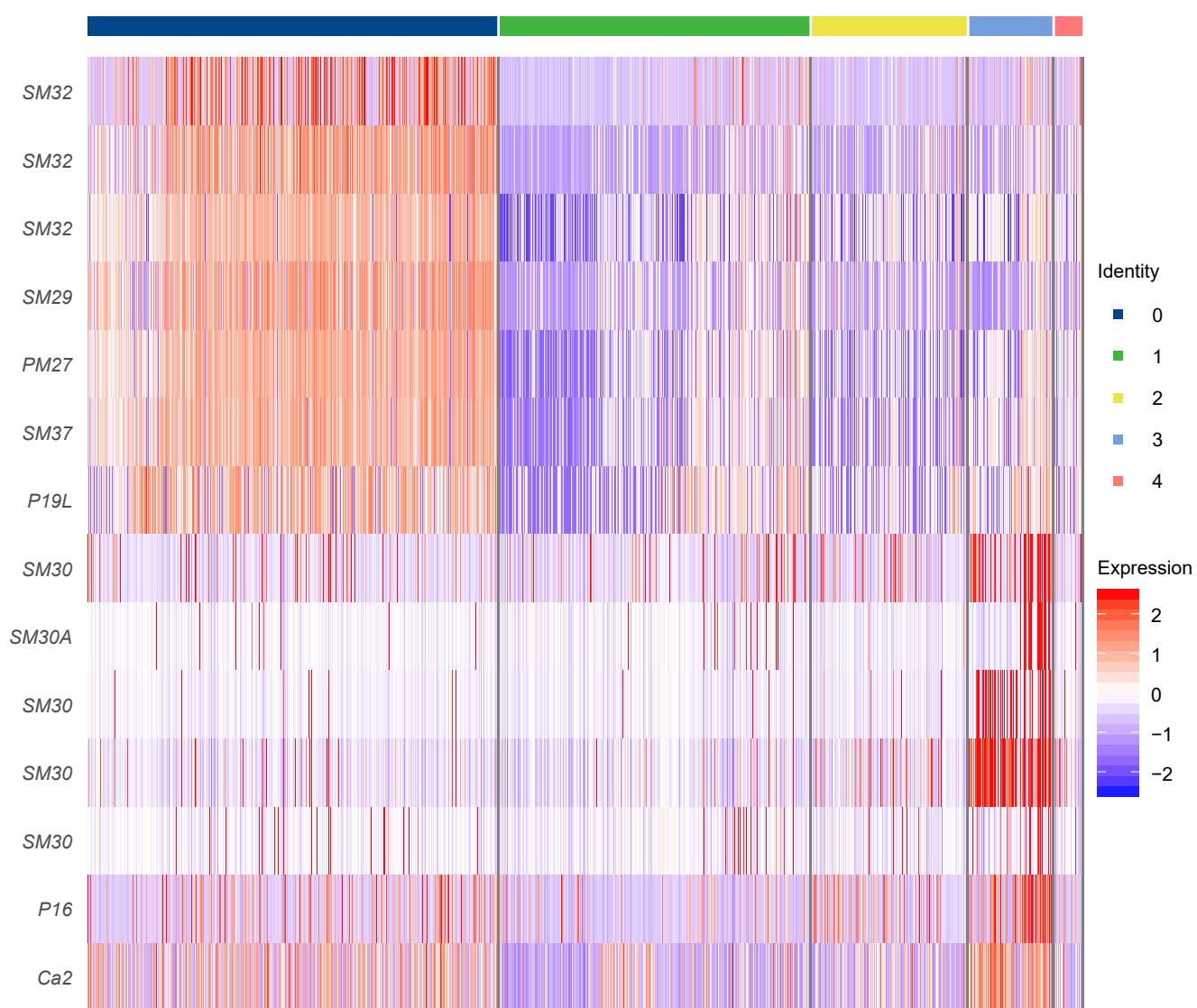

Supplement: Supplementary file 1 [file ijms-26-01059-s001.zip › Supplementary File S6. Heatmap of biomineralizing factor expression profiles in the SC subclusters.pdf]
